# Impact of China’s National Volume-Based Drug Procurement: A Multilevel Interrupted Time Series Analysis on Medical Expenditures in Hypertensive Patients

**DOI:** 10.34172/ijhpm.8540

**Published:** 2025-05-25

**Authors:** Yunxiang Huang, Yan Ren, Yuanjin Zhang, Yulong Jia, Qianrui Li, Minghong Yao, Yuning Wang, Fan Mei, Kang Zou, Huangang Hu, Jing Tan, Xin Sun

**Affiliations:** ^1^Institute of Integrated Traditional Chinese and Western Medicine, and Chinese Evidence-based Medicine Center, West China Hospital, Sichuan University, Chengdu, China.; ^2^NMPA Key Laboratory for Real World Data Research and Evaluation in Hainan, Chengdu, China.; ^3^Sichuan Center of Technology Innovation for Real World Data, Chengdu, China.; ^4^Department of Nuclear Medicine, West China Hospital of Sichuan University, Chengdu, China.; ^5^Tianjin Healthcare and Medical Big Data Co., Ltd, Tianjin, China.; ^6^Department of Epidemiology and Biostatistics, West China School of Public Health, Sichuan University, Chengdu, China.

**Keywords:** Drug Procurement, Volume-Based, Medical Expenditures, China

## Abstract

**Background:**

The National Volume-Based Procurement (NVBP), implemented in China in 2019, aims to reduce patients’ economic burden by lowering drug prices and promoting the use of NVBP drugs in public hospitals. We evaluated the impact of NVBP on medical expenditures among hypertensive patients, analyzing both the overall impact and variations in policy effects across individual hospitals.

**Methods:**

Using medical records from 1.17 million hypertensive patients across 82 hospitals in Tianjin (2017-2021), we conducted an interrupted time series analysis to assess expenditure changes among hypertensive patients for the treatment of hypertension, dyslipidaemia, type 2 diabetes, and chronic ischemic heart disease (IHD). Multilevel model was employed to estimate the overall impact and hospital-specific variations in policy effects.

**Results:**

NVBP implementation significantly reduced per-visit outpatient expenditures among hypertensive patients for the treatment of hypertension (-15.61%), dyslipidaemia (-25.77%), and diabetes (-17.59%) by lowering drug expenditures. Although drug expenditures for chronic IHD decreased, non-drug expenditures increased, leading to no significant change in total expenditures for chronic IHD (-8.97%). For inpatient expenditures, no significant changes in total per-admission expenditures were observed for chronic IHD or diabetes hospitalizations. Drug expenditures for diabetes decreased significantly, but diagnostic expenditures increased, while no significant change was found in chronic IHD drug expenditures. At the individual hospital level, significant variations in policy effects were observed. Despite the overall decrease in outpatient expenditures for the treatment of hypertension, dyslipidaemia, and diabetes, only 45.6%, 67.2%, and 46.3% of hospitals, respectively, showed significant decreases, while the remainder exhibited either non-significant changes or increases.

**Conclusion:**

NVBP effectively reduced outpatient expenditures among hypertensive patients for the treatment of hypertension, dyslipidaemia, and diabetes, suggesting its potential to alleviate patients’ economic burdens. However, the increases in non-drug expenditures and substantial variations in policy effects across hospitals highlight a room for further improvement in policy implementation and overall effectiveness.

## Background

**Key Messages**

**Implications for policy makers**

- By lowering drug prices through centralized procurement and promoting the use of lower-priced, centralized procured drugs in public hospitals, the National Volume-Based Procurement (NVBP) program in China may serve as an effective strategy for reducing patients’ economic burdens, as evidenced by significant reductions in total outpatient expenditures among hypertensive patients for the treatment of hypertension, dyslipidaemia, and diabetes after its implementation.
- Despite reductions in drug expenditures, significant increases in non-drug expenditures were observed for the treatment of several conditions in both outpatient and inpatient settings. Dynamic monitoring is essential to ensure that the cost savings from reduced drug expenditures under the NVBP are not offset by the increased spending on unnecessary drugs or non-drug services.
- Although the NVBP resulted in an overall reduction in outpatient expenditures, nearly half of hospitals did not show significant reductions. This suggests that while the NVBP has been effective, there is still a room for improvement. To further enhance the policy’s overall effectiveness, it is crucial to investigate the reasons behind these variations across different hospitals.

**Implications for the public**

The National Volume-Based Procurement (NVBP) program was implemented in China to alleviate the economic burden on patients by lowering drug prices through centralized procurement and promoting the use of NVBP drugs in public hospitals. By using medical records from a cohort of 1.17 million hypertensive patients over five years, we found a significant 15.61% reduction in total outpatient expenditures per visit for hypertension treatment. Significant reductions were also observed for related treatment of comorbidities among hypertensive patients, including dyslipidaemia (-25.77%) and diabetes (-17.59%). These results highlight the NVBP’s potential to benefit patients with chronic conditions who require long-term, combined medications. However, increases in non-drug expenditures per medical visit might partially offset the policy’s overall effectiveness. Furthermore, while outpatient expenditures for hypertension, dyslipidaemia, and diabetes treatments decreased overall, nearly half of the hospitals did not show similarly significant reductions. If these hospitals can achieve favorable effects in the future, the financial burden on patients could be further reduced.

The soaring drug expenditures place a heavy economic burden on patients and healthcare systems worldwide.^1^ Centralized drug procurement, also known as pooled or volume-based procurement, consolidates resources from multiple purchasing authorities to leverage economies of scale and scope, and has been an important policy to contain the rise in drug expenditures in countries with different income levels.^2^ Examples include national centralized procurement systems for public hospitals in Denmark and Norway,^3^ regional purchasing bodies in Italy,^4^ and specialized national programs (eg, for cancer) in India.^5^

 In 2019, the Chinese government launched a National Volume-Based Procurement (NVBP) program to lower drug prices and reduce the economic burden on patients.^6^ Under this initiative, all public hospitals are required to participate in the national procurement of pre-specified drugs, with procurement volumes set at 60%–80% of their previous annual usage levels. Moreover, hospitals are mandatory to prioritize prescribing NVBP drugs over more expensive, non-bid-winning alternatives. As of 2022, seven rounds of NVBP have been carried out, covering 294 drugs that are commonly used in clinical settings, with prices decreasing by an average of 53%, saving the healthcare system an estimated more than ¥ 260 billion.^7^

 While there is a well-established body of literature documenting the NVBP’s effectiveness in reducing drug prices and procurement expenditures,^8,9^ less is known about its impact on medical expenditures for patients. Intuitively, reducing drug prices should lower drug expenditures and, consequently, overall medical expenditures for patients. However, according to the standard model of physician behavior proposed by McGuire and Pauly,^10^ there is a possibility that physicians may respond to price reductions by increasing the provision of services or products with higher profit margins. In a previous study on hospital drug purchasing behavior,^11^ Chen et al found that although the NVBP successfully reduced expenditures on policy-targeted drugs, total drug expenditures were not effectively controlled due to increased use of non-targeted higher-priced alternatives. Given the potential cost-shifting effect, the actual impact of NVBP on medical expenditures remains uncertain, and empirical studies are needed.

 Existing studies on centralized drug procurement, whether from developed or developing countries, have primarily focused on drug prices or procurement expenditures following policy implementation, with an emphasis on the cost savings for purchasing authorities.^9,12^ Although several studies in China have explored the impact of NVBP on medical expenditures for patients, the majority of them were based on data from a single hospital or analyzed expenditures related to a single medical condition,^13-15^ thereby largely restricting their generalizability. Moreover, most studies have concentrated on the first round of the NVBP, with less attention given to the longer-term effects of subsequent rounds.^13,14,16^ As the program evolves, further research is needed to provide a more comprehensive evaluation.

 In addition, no study has yet explored the varying policy effects across individual hospitals. As the primary implementers of the NVBP, hospitals play a critical role in determining the policy’s effectiveness. The overall success of the program largely depends on how each hospital executes it. Quantifying the varying policy effects across individual hospitals could provide policy-makers with more detailed insights into the policy’s implementation effectiveness across institutions, helping identify areas for improvement and ultimately enhancing the policy’s overall impact.

 In this study, we evaluated the impact of NVBP implementation on patient medical expenditures, analyzing both the overall impact and the variation in policy effects across individual hospitals. Using electronic medical records data from a cohort of 1.17 million hypertension patients across 82 public hospitals in Tianjin between January 2017 and December 2021, we assessed the expenditure changes among hypertensive patients for the treatment of four common medical conditions: essential hypertension, dyslipidaemia, type 2 diabetes, and chronic ischemic heart disease (IHD). Hypertensive patients were selected to evaluate the impact of NVBP on patient medical expenditures because the antihypertensive medications constitute the majority drugs included in the NVBP procurement list. By analyzing expenditures for hypertension treatment alongside three common comorbidities among patients with hypertension, we aimed to provide a more comprehensive assessment of the NVBP’s impact on patients’ economic burdens.

## Methods

###  National Volume-Based Drug Procurement Overview

 During the study period from January 2017 to December 2021, five rounds of NVBP were conducted. The first pilot round, implemented in April 2019, involved 11 cities, including Beijing, Shanghai, and Tianjin, which were grouped together in a tender process to bulk-buy 25 drugs.^17^ The second round, conducted one year after the pilot in May 2020, expanded to a nationwide scale, with all public hospitals across the country participating in the centralized procurement of 32 drugs.^18^ The third, fourth, and fifth rounds followed, procuring 55, 44, and 63 drugs, respectively, with the implementation intervals progressively shortened to 6 months, 6 months, and 4 months. In total, 218 drugs were included in the first five rounds of NVBP, of which only 12 were branded, while the majority (94.4%, 206/218) were generic. Among the 218 drugs, 44 (20%) were related to cardiovascular and diabetes therapies (See Supplementary file 1 for NVBP overview).

###  Study Setting

 We set our study in Tianjin, one of the pilot cities to launch the NVBP program in 2019.^17^ During the pilot initiative, all public hospitals in this region had participated in the centralized drug procurement and utilization. The successful pilot implementation in Tianjin served as a model for the program’s subsequent nationwide expansion.^18^ The findings from evaluating the NVBP program in Tianjin therefore might, in some extent, be representative of other regions.

###  Data Source

 We used data from the Tianjin Health Care Big Data Platform, an integrated health information system administered by the Tianjin Municipal Health Commission to manage the healthcare data for local residents. As of 2021, this database has collected electronic medical records from 82 hospitals—43 tertiary and 39 secondary—covering nearly 26.1 million patients. The medical records data in data platform includes clinical information, prescriptions, lab results, vital signs, body measurements, diagnoses, procedures, and cost details at the level of individual patient visits.

###  Study Population

 Based on the Tianjin Health Care Big Data Platform, we constructed a large cohort database of hypertension that includes longitudinal patient-level medical records of 1 172 280 hypertensive patients over seven years, from January 1, 2015 to December 31, 2021. Patients aged 18 years or older, with a first diagnosis of hypertension between January 2015 and December 2021, were identified from the data platform using ICD-10-CM codes (International Classification of Diseases, Tenth Revision, Clinical Modification; I10) and diagnostic text. To be included in the cohort, patients had to have at least two outpatient diagnoses or one hospital discharge diagnosis of hypertension during the study period.^19^ All medical records of the study population from 2015 to 2021 were extracted from the data platform in 2022.

###  Sample Selection

 Using ICD-10-CM codes, we identified medical visit records for four targeted medical conditions among hypertensive patients in cohort: hypertension (I10), dyslipidaemia (E78), chronic IHD (I25), and diabetes (E11), from the time of their initial hypertension diagnosis until the end of the study period. These conditions were selected based on two major considerations. First, hypertension frequently co-occurs with other chronic disease,^20^ and hypertensive patients incur medical expenditures not only for managing their primary hypertension but also for treating these common comorbidities. Second, the medications used to treat these conditions are largely covered by the NVBP, making them appropriate indicators for evaluating the policy’s effectiveness (NVBP-covered medications for these medical conditions; see Supplementary file 1).

###  Outcome Measures

 The outcome measures were monthly mean expenditures per-patient-visit for the treatment of four targeted medical conditions: hypertension, dyslipidaemia, chronic IHD, and diabetes, including both outpatient and inpatient expenditures. For outpatient expenditures, we analyzed three categories: (*i*) total expenditures, (*ii*) drug expenditures, and (*iii*) non-drug-related expenditures. For inpatient expenditures, we analyzed six categories: (*i*) total expenditures, (*ii*) out-of-pocket (OOP) expenditures, (*iii*) drug expenditures, (*iv*) diagnostic expenditures, (*v*) treat expenditures, and (*vi*) consumable expenditures.

 OOP expenditures refer to the portion of total expenditures directly paid by patients after health insurance reimbursement. Diagnostic expenditures include fees for various diagnostic tests, such as pathological, laboratory, imaging, and clinical tests. Treat expenditures include fees for both surgical and non-surgical treatments, reflecting the medical procedures performed by physicians. Consumable expenditures refer to the cost of single-use medical materials.

 We specifically analyzed three sub-components of non-drug-related expenditures (ie, diagnostic, treat, and consumable) in inpatient care because previous studies on pharmaceutical policies have highlighted the potential for cost-shifting in these categories.^21^ However, for outpatient expenditures, we did not further differentiate these sub-components, as the majority of outpatient expenditures were drug-related. Additional stratification of non-drug expenditures would result in a large proportion of zero values, leading to unreliable analysis. Since outpatient expenditures were almost entirely paid by patients without reimbursement, OOP expenditures were largely equivalent to total expenditures. Therefore, we did not conduct a separate analysis of OOP expenditures for outpatient care.

 To ensure expenditures were correctly attributed to the relevant clinical contexts, for outpatient expenditures, we included only records with a single diagnosis of hypertension, dyslipidaemia, chronic IHD, or diabetes in the analysis. For inpatient expenditures, we included records with a principal discharge diagnosis of either diabetes or chronic IHD, excluding those with both diagnoses as principal (n = 173). Since hospitalizations for hypertension or dyslipidaemia are often complicated by severe or acute comorbidities, expenditures related to antihypertensive or lipid-lowering therapies during hospitalization are likely to represent only a small portion of the total inpatient expenditures. To mitigate potential misclassification bias, we excluded hypertension and dyslipidaemia from the inpatient expenditure analyses.

 To ensure the validity of the expenditure measures, several data adjustments were applied. Expenditure values below the 5th percentile and above the 95th percentile for each expenditure outcome were excluded to minimize the influence of extreme outliers on mean estimation. To ensure comparability of drug expenditures per medical visit before and after the introduction of the long-term prescription policy in January 2020, drug expenditures for each outpatient visit across study years were adjusted to a 14-day prescription length.^22^ To minimize potential confounding effects from the implementation of the zero-markup drug policy in December 2016,^23^ this study included only expenditure data from January 2017 onward, although earlier data were available. Additionally, four months of expenditure data (January 2020 to April 2020) were excluded due to disruptions in healthcare services caused by the COVID-19 outbreak.^24^ All expenditures during the study period were adjusted for inflation to 2021 Chinese Yuan (RMB).^25^

###  Study Design 

 An interrupted time series design was employed to evaluate the impact of NVBP on medical expenditures for patients by comparing the level and trend of per-patient-visit expenditures before and after the NVBP implementation.^26^ We assumed that expenditures would decrease immediately, without any delays, as lower-priced drugs became available to patients shortly after the policy’s implementation. As four additional NVBP rounds were carried out consecutively in the years after the pilot phase, the number of lower-priced NVBP drugs gradually increased. This gradual expansion may have created a cumulative effect, potentially altering expenditure trends in the post-NVBP period compared to the pre-NVBP period.

 To identify the most appropriate impact model that accounts for the gradual rollout of NVBP rounds, we conducted an exploratory analysis of per-visit outpatient drug expenditures changes. The analysis revealed a two-segment change in expenditures following the NVBP implementation, with the second segment occurring at the NVBP expansion round (Study design consideration; see Supplementary file 2).

 Finally, we segmented the study period into three separate periods: pre-NVBP (January 2017 through March 2019), NVBP polit (April 2019 through December 2019), and NVBP expansion (May 2020 through December 2021). Since anti-diabetic drugs were not included until the expansion of NVBP, we analyzed expenditures related to diabetes treatments for two separate periods: pre-NVBP expansion (January 2017 through December 2019), and NVBP expansion (May 2020 through December 2021).

###  Statistical Analysis

 In the main analysis, the monthly expenditures per-patient-visit were averaged at the hospital level, with monthly expenditures (level 1) nested within hospitals (level 2). A two-level regression model was employed to estimate the overall level and trend changes in expenditures following the NVBP pilot and NVBP expansion periods.^27^ To quantify the varying expenditure changes across individual hospitals, hospital-specific random residual error terms were included in the two-level regression model, allowing for different intercept coefficients and different slope coefficients for level changes in expenditures post-NVBP across different hospitals (Analytic model specification; see Supplementary file 3). A likelihood ratio test was conducted on the variance component of the two-level regression model to assess the statistical significance of variations in expenditure changes between hospitals.

 Using the coefficients estimated from the two-level regression model, we computed two types of predicted expenditures to estimate: (*i*) the overall relative change in expenditures; and (*ii*) the varying relative changes in expenditures across individual hospitals.^28^ To estimate the overall relative change in expenditures, we computed mean predicted expenditures across all hospitals, setting the variance of hospital-level residual errors to zero. To estimate the varying relative changes in expenditures across individual hospitals, we computed hospital-specific predicted expenditures based on the mean predicted expenditures, conditional on the hospital-specific random residual errors.

 The relative change in expenditures following NVBP implementation was expressed as the percentage difference between the average predicted expenditures over the entire post-NVBP period under the factual scenario and counterfactual scenario, with the counterfactual scenario serving as the reference (Calculation equation; see Supplementary file 3). This measure reflects the percentage change in expenditures with the implementation of NVBP, compared to the expected expenditures without NVBP.^29^ To estimate the 95% confidence interval (CI) for the relative changes, a Monte Carlo simulation (n = 10 000) was performed, using the 2.5th and 97.5th percentiles of the simulated values to define the 95% CI.

 Bases on the 95% CI of relative change in expenditures, hospitals were categorized into three groups: (*i*) those with significant expenditure decreases (where the entire of 95% CI was below zero); (*ii*) those with non-significant changes (where the 95% CI included zero); and (*iii*) those with significant expenditure increases (where the entire of 95% CI was above zero).^30^ For each group, we summarized the number and proportion of hospitals, as well as the median and range of expenditure changes.

 To examine the robustness of our findings to modeling specification, we added a “aggregate-level” segmented regression as sensitivity analysis. In this sensitivity analysis, the per-patient-visit expenditures within a given month were averaged at the highest population level. The generalized least squares model was used to estimate the level and trend changes in expenditures.^31^ Durbin-Watson test was conducted to identify the autocorrelation, and the autoregressive-moving average correlation structure was added into the generalized least squares models to control the autocorrelation if needed.^32^

 We prespecified five covariates for all regression models: the monthly proportion of males, mean age, mean number of unique medications, mean number of comorbid diagnoses and calendar months to control the potential time-varying confounders and seasonality of time series data.^26,33^ A two-sided *P* value of less than 0.05 was considered to indicate significance. All statistical analyses were performed using R software version 4.3.0.

## Results

 The cohort characteristics of 1 172 280 hypertensive patients before and after the NVBP are detailed in Supplementary file 4. This study analyzed 3 225 890 outpatient records and 68 491 inpatient records over a 5-year period (January 2017–December 2021) for hypertensive patients in the cohort.For outpatient expenditures, we included 1 827 617 visits for hypertension, 234 076 for dyslipidaemia, 475 736 for chronic IHD, and 688 461 for diabetes. For inpatient expenditures, we analyzed 28 729 admissions for chronic IHD and 39 762 for diabetes (Table S2, Supplementary file 4). Mean outpatient expenditure per visit and inpatient expenditures per admission before and after the NVBP are presented in Tables S3-S4 in Supplementary file 4.

###  Overall Impact of NVBP Implementation on Medical Expenditures

####  Outpatient Expenditures

**Figure 1:**
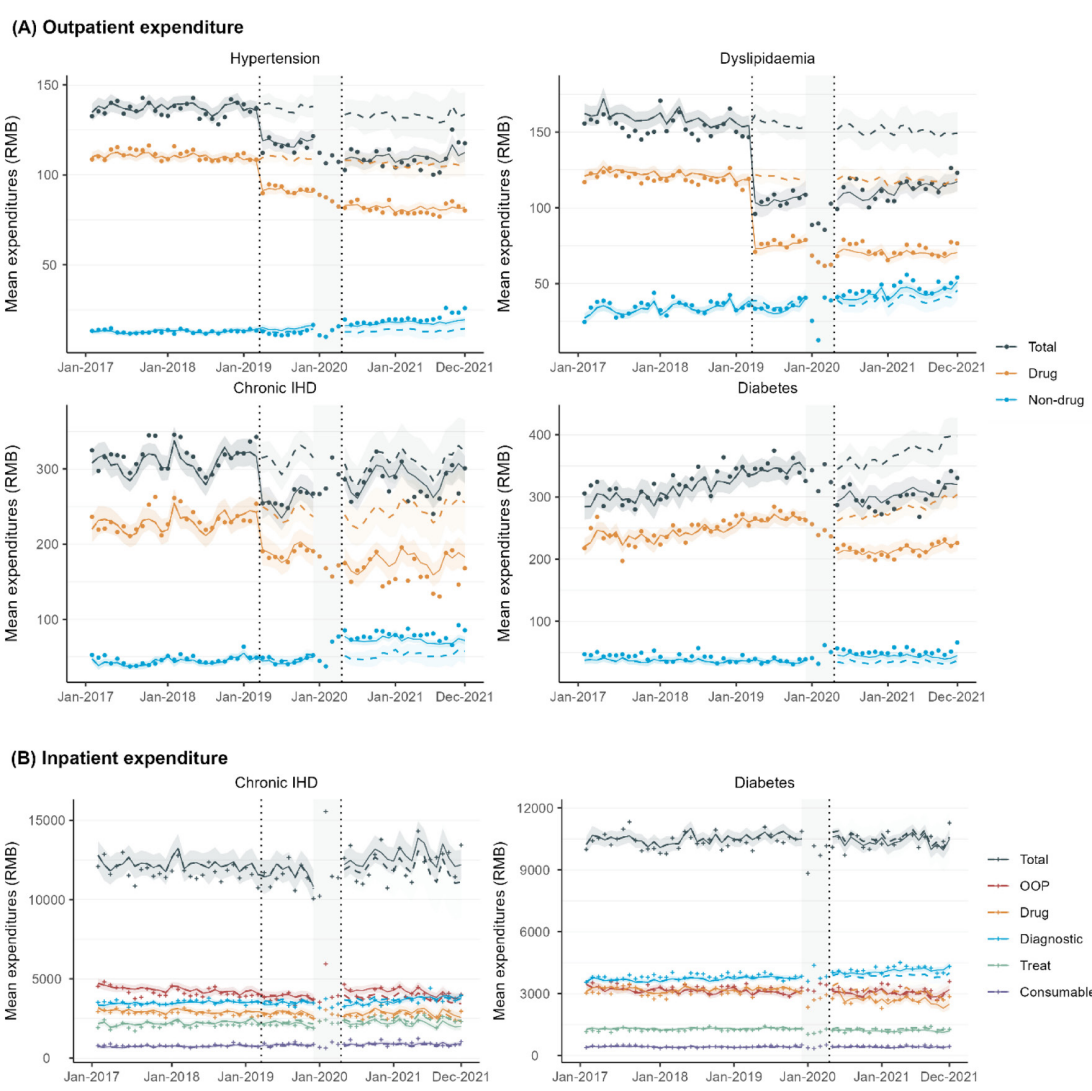
Interrupted Time Series Plots of Medical Expenditure Changes. This figure presents the monthly mean expenditures per outpatient visit (Panel A) and per inpatient admission (Panel B) before and after the NVBP implementation. Dots represent mean expenditures per visit or admission, calculated as the average across hospitals. The solid lines show the model-fitted observed expenditures (factual), estimated using fixed-effect terms from multilevel models, while the dashed lines illustrate the model-fitted expected expenditures (counterfactual) under the assumption that the NVBP had not been implemented. Shaded areas around the lines indicate 95% CIs for the fitted values. The first segment corresponds to the launch of the NVBP pilot in April 2019, and the second segment marks the NVBP expansion in May 2020. Notably, drugs for type 2 diabetes were not included in the NVBP pilot but were added after the NVBP expansion round in April 2020. The gray rectangular shaded area represents the period of the COVID-19 outbreak. Abbreviations: NVBP, National Volume-Based Procurement; IHD, ischaemic heart disease; OOP, out-of-pocket; CI, confidence interval.

 Segmented regression models showed that there was a significant and immediate reduction in the level of total expenditures per outpatient visit across four medical condition following the NVBP: hypertension (-20.24; 95% CI, -27.59 to -12.9), dyslipidaemia (-59.38; 95% CI, -72.89 to -45.86), chronic IHD (-61.64; 95% CI, -92.73 to -30.55), and diabetes (-49.95; 95% CI, -78.26 to -21.64), and these reductions were primarily driven by the decreases in drug expenditures (Figure 1, Panel A; Table 1). Additional level reductions in per-visit drug expenditures were observed for hypertension and dyslipidaemia during the NVBP expansion period. In contrast, compared to the period of NVBP pilot, the level of per-visit non-drug expenditures for chronic IHD and diabetes showed significant increases during the NVBP expansion period.

**Table 1:** Segmented Regressions on Level and Trend Changes in Medical Expenditures

	**Intercept** **β**_0_** (95% CI)**	**Trend Before NVBP** **β**_1_** (95% CI)**	**NVBP Pilot (April 2019–January 2020)**		**NVBP Expansion (May 2020–December 2021)**	
			**Level Change** **β**_2_** (95% CI)**	**Trend Change** **β**_3_** (95% CI)**	**Level Change** **β**_4_** (95% CI)**	**Trend Change** **β**_5_** (95% CI)**
**Outpatient Expenditures**						
Hypertension						
Total	135.82 (121.2 to 150.44)	-0.09 (-0.26 to 0.09)	-20.24 (-27.59 to -12.9)^***^	0.21 (-0.76 to 1.17)	-6.67 (-19.22 to 5.88)	-0.02 (-1 to 0.97)
Drug	110.49 (102.98 to 118)	-0.13 (-0.24 to -0.02)^*^	-18.37 (-24.12 to -12.61)^***^	0 (-0.59 to 0.6)	-6.66 (-12.24 to -1.08)^*^	0.05 (-0.56 to 0.65)
Non-drug	13.05 (8.27 to 17.84)	0.02 (-0.03 to 0.06)	1.16 (-4 to 6.33)	0.12 (-0.12 to 0.36)	1.2 (-5.46 to 7.87)	-0.04 (-0.29 to 0.21)
Dyslipidaemia						
Total	161.26 (146.45 to 176.08)	-0.2 (-0.41 to 0.02)	-59.38 (-72.89 to -45.86)^***^	1.78 (0.59 to 2.98)^**^	-3.9 (-15.56 to 7.75)	-1.03 (-2.25 to 0.2)
Drug	120.18 (112.64 to 127.73)	-0.11 (-0.23 to 0.02)	-49.63 (-57.89 to -41.38)^***^	0.94 (0.24 to 1.64)^**^	-7.29 (-13.58 to -0.99)^*^	-0.93 (-1.65 to -0.22)^*^
Non-drug	26.81 (19.36 to 34.26)	0.19 (0.06 to 0.33)^**^	-5.18 (-10.78 to 0.42)	0.32 (-0.41 to 1.04)	5.82 (-0.27 to 11.91)	-0.2 (-0.94 to 0.54)
Chronic IHD						
Total	297.02 (247.08 to 346.95)	0.28 (-0.47 to 1.03)	-61.64 (-92.73 to -30.55)^***^	0.91 (-3.21 to 5.02)	37.42 (2.91 to 71.94)^*^	-1.31 (-5.52 to 2.9)
Drug	209.63 (168.35 to 250.91)	0.7 (0.13 to 1.27)^*^	-58.68 (-83.47 to -33.88)^***^	1.47 (-1.68 to 4.62)	-14.42 (-39.51 to 10.68)	-2.11 (-5.34 to 1.11)
Non-drug	46.65 (35.65 to 57.66)	0.3 (0.09 to 0.51)^**^	-4.86 (-13.03 to 3.31)	0.19 (-0.95 to 1.32)	29.36 (19.2 to 39.52)^***^	-0.79 (-1.95 to 0.37)
Diabetes^a^						
Total	287.31 (250.62 to 324)	1.8 (1.29 to 2.31)^***^	-	-	-49.95 (-78.26 to -21.64)^***^	-1.38 (-2.59 to -0.18)^*^
Drug	228.25 (208.77 to 247.74)	1.14 (0.77 to 1.5)^***^	-	-	-50.97 (-69.91 to -32.03)^***^	-1.28 (-2.15 to -0.42)^**^
Non-drug	36.33 (25.24 to 47.41)	-0.04 (-0.2 to 0.12)	-	-	11.34 (2.49 to 20.2)^*^	-0.17 (-0.53 to 0.19)
**Inpatient Expenditures**						
Chronic IHD						
Total	12 213.66 (10 903.53 to 13 523.79)	-0.08 (-27.26 to 27.11)	-101.67 (-1067.58 to 864.24)	24.85 (-127.92 to 177.62)	153.9 (-1005.8 to 1313.6)	15.82 (-143.93 to 175.57)
OOP	4629.8 (4042.38 to 5217.21)	-14.46 (-25.66 to -3.27)^*^	-116.05 (-516.05 to 283.95)	21.95 (-41.15 to 85.04)	384.75 (-96 to 865.49)	-30.09 (-95.9 to 35.72)
Drug	2982.64 (2517.49 to 3447.78)	-3.25 (-11.97 to 5.47)	59.84 (-252.24 to 371.91)	10.88 (-38.48 to 60.24)	-169.15 (-587.18 to 248.87)	1.87 (-49.75 to 53.5)
Diagnostic	3256.31 (2904.29 to 3608.33)	9.06 (3.5 to 14.62)^**^	-259.58 (-456.99 to -62.17)^**^	25.54 (-5.66 to 56.74)	-360.43 (-598.41 to -122.45)^**^	2.49 (-30.07 to 35.04)
Treat	2089.6 (1745.33 to 2433.87)	9.64 (1.94 to 17.34)^*^	68.65 (-208.09 to 345.38)	-36.79 (-80.59 to 7.02)	120.1 (-166.89 to 407.09)	24.2 (-21.36 to 69.76)
Consumable	702.09 (502.39 to 901.78)	2.61 (-1.51 to 6.74)	109.31 (-37.78 to 256.4)	-7.01 (-30.26 to 16.23)	0.12 (-173.23 to 173.47)	8.93 (-15.33 to 33.18)
Diabetes^a^						
Total	10 454.2 (9712.06 to 11 196.34)	-2.1 (-11.15 to 6.95)	-	-	-240.65 (-819.89 to 338.58)	7.04 (-19.69 to 33.76)
OOP	3484.99 (3231.55 to 3738.43)	-7.15 (-11.37 to -2.93)^***^	-	-	-10.03 (-213.5 to 193.44)	6.02 (-6.55 to 18.59)
Drug	3281.62 (2882.82 to 3680.43)	-6.02 (-10.64 to -1.4)^*^	-	-	-403.96 (-709.81 to -98.11)^**^	-2.21 (-16.02 to 11.6)
Diagnostic	3605.83 (3272.09 to 3939.56)	3.85 (0.28 to 7.42)^*^	-	-	90.31 (-98.3 to 278.93)	13.87 (3.34 to 24.41)^**^
Treat	1295.29 (1094.41 to 1496.18)	-1.01 (-3.21 to 1.18)	-	-	-90.09 (-252 to 71.82)	-0.29 (-6.81 to 6.23)
Consumable	418.89 (344.45 to 493.34)	-1.35 (-2.22 to -0.48)^**^	-	-	2.09 (-46.58 to 50.76)	1.39 (-1.21 to 4)

Abbreviations: NVBP, National Volume-Based Procurement; IHD, ischaemic heart disease; OOP, out-of-pocket; CI, confidence level. ^a^ Drugs for type 2 diabetes were not included in the NVBP pilot until the NVBP expansion in April 2020; ^***^ *P* < .001; ^**^ *P* < .01; ^*^ *P* < .05.

**Figure 2:**
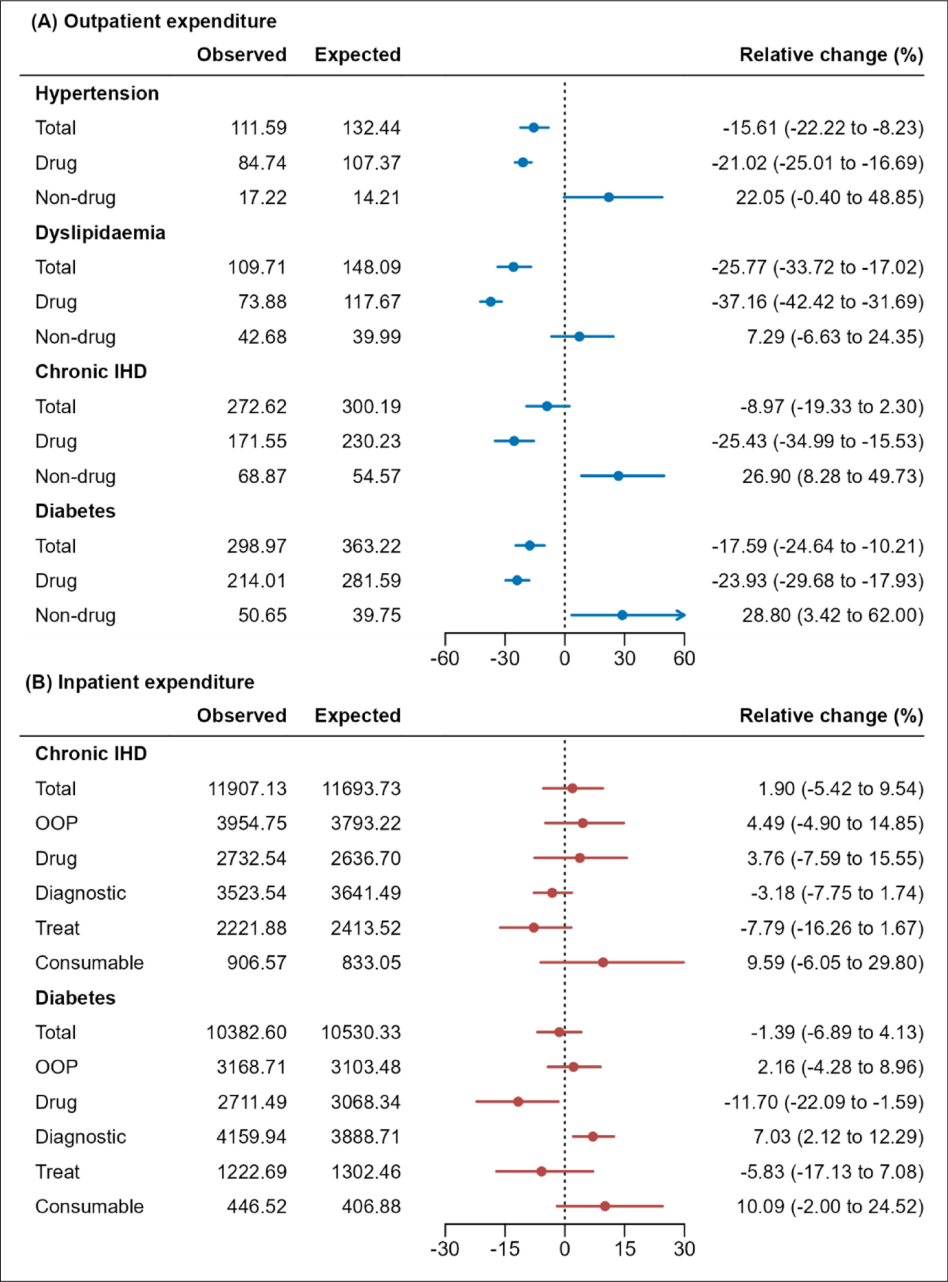
Overall Relative Changes (%) in Medical Expenditures. This figure illustrates the relative changes in outpatient (Panel A) and inpatient (Panel B) expenditures after the NVBP. Observed expenditures represent the average monthly expenditures per visit or admission during the entire NVBP period (factual), while expected expenditures correspond to the average monthly expenditures per visit or admission during the same period, assuming the NVBP had not been implemented (counterfactual). The relative change is expressed as the percentage difference between observed and expected expenditures, relative to the expected expenditures. Squares denote point estimates of the relative change, and error bars indicate 95% CIs, calculated using 10 000 simulations. Abbreviations: NVBP, National Volume-Based Procurement; IHD, ischaemic heart disease; OOP, out-of-pocket; CI, confidence interval.

 Compared with the expected expenditures without NVBP, the relative changes in per-visit drug expenditures during the post-NVBP period showed significant decreases across all four medical conditions (Figure 2, Panel A). However, the per-visit non-drug expenditures increased significantly by 26.9% for chronic IHD and 28.8% for diabetes. Overall, the total expenditures per visit declined significantly by 15.6% for hypertension, 25.77% for dyslipidaemia, and 17.59% for diabetes. For chronic IHD, a reduction of 8.79% in total expenditures per visit was observed, but this decrease was not statistically significant (95% CI, -19.33% to 2.30%).

####  Inpatient Expenditures

 Segmented regression models showed non-significant changes in both level and trend of total or OOP expenditures per admission for chronic IHD and diabetes hospitalization (Table 1; Figure 1, Panel B). No significant level or trend changes in drug expenditures per admission were observed for chronic IHD hospitalization after the NVBP. In contrast, the level of drug expenditures per admission for diabetes decreased significantly after NVBP implementation (-403.96; 95% CI, -709.81 to -98.11), while the trend in diagnostic expenditures showed a significant increase (13.87; 95% CI, 3.34 to 24.41). No significant changes in treat or consumable expenditures were observed for either condition.

 Overall, compared with the expected expenditure assuming that the NVBP had not implemented, the relative changes in total expenditures per admission and individual expenditure components for chronic IHD hospitalizations were not statistically significant (Figure 2, Panel B). For diabetes, while average drug expenditures per admission decreased by 11.70% (95% CI, -22.09% to -1.59%), diagnostic expenditures per admission increased by 7.03% (95% CI, 2.12% to 12.29%), resulting in no significant change in total expenditures per admission for diabetes hospitalization.

###  Varying Policy Effects Across Individual Hospitals

 The likelihood ratio test on the variance component of the multilevel models revealed a *P *value of less than.001 for all expenditure outcome models, indicating statistically significant variation in expenditure changes between hospitals.

####  Outpatient Expenditures

**Figure 3:**
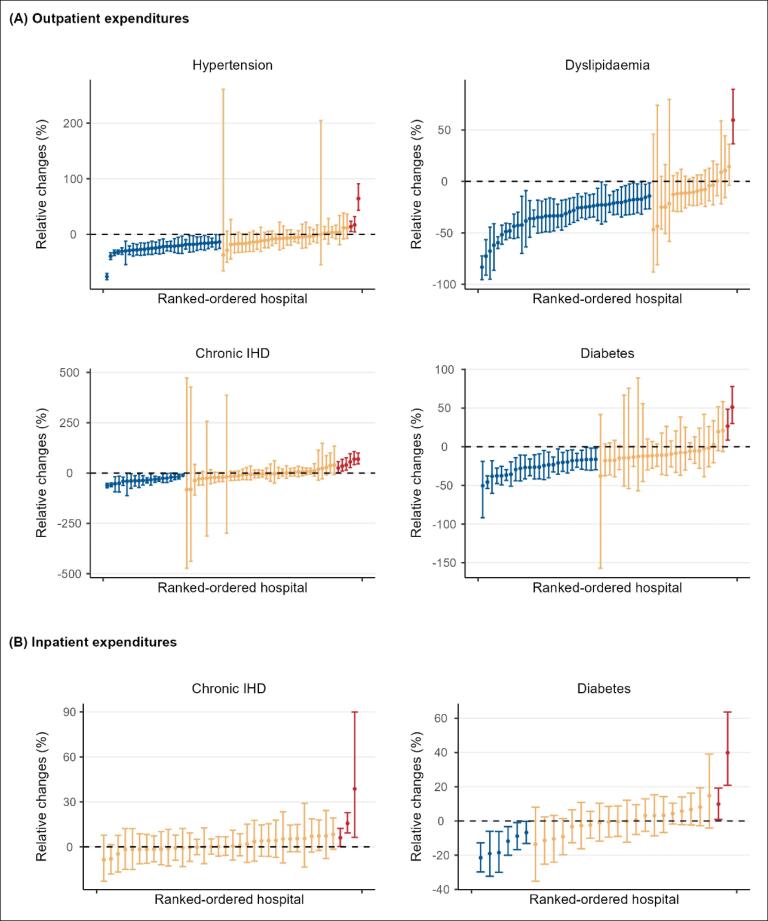
Distribution of Relative Changes in Medical Expenditures Across Hospitals. This figure shows the distribution of relative changes in total expenditures for outpatient visit (Panel A) and inpatient admission (Panel B) across hospitals following the implementation of the NVBP. Each dot represents the relative change for a hospital, with error bars indicating the 95% CIs. Blue indicates a significant decrease (95% CI entirely below zero), yellow indicates no significant change (95% CI includes zero), and red indicates a significant increase (95% CI entirely above zero). Abbreviations: NVBP, National Volume-Based Procurement; IHD, ischaemic heart disease; CI, confidence interval.

 The relative changes in total expenditures per outpatient visit among hospitals ranged from -75.95% to 64.43% for hypertension, -83.3% to 59.5% for dyslipidaemia, -62.0% to 69.4% for chronic IHD, and -50.4% to 51.1% for diabetes (Table 2). Statistically significant reductions in expenditures were observed in 45.6% (31/68) of hospitals for hypertension, 67.2% (43/64) for dyslipidaemia, 31.2% (20/64) for chronic IHD, and 46.3% (25/54) for diabetes. Non-significant reductions in expenditures were reported in 50.0% (34/68) of hospitals for hypertension, 31.2% (20/64) for dyslipidaemia, 59.4% (38/64) for chronic IHD, and 50.0% (27/54) for diabetes. A very small proportion of hospitals exhibiting significant increases in expenditures (Figure 3, Panel A).

**Table 2:** Hospital-Level Variation in Relative Changes in Medical Expenditures

	**Hospitals**^a^ **No. **	**Significant Decrease **		**Non-significant Change **		**Significant Increase **	
		**Hospitals** **No. (%)**	**Relative Change (%)** **Median (Range)**	**Hospitals** **No. (%)**	**Relative Change (%)** **Median (Range)**	**Hospitals** **No. (%)**	**Relative Change (%)** **Median (Range)**
**Outpatient Expenditures**							
Hypertension							
Total	68	31 (45.6%)	-22.06 (-75.94, -13.56)	34 (50%)	-6.99 (-37.08, 11.71)	3 (4.4%)	17.01 (13.94, 64.43)
Drug	68	47 (69.1%)	-21.81 (-52.39, -12.39)	21 (30.9%)	-7.07 (-23.42, 9.6)	0	-
Non-drug	72	2 (2.8%)	-34.35 (-46.58, -22.11)	65 (90.3%)	1.55 (-16 721.39, 1503.45)	5 (6.9%)	143.17 (18.16, 1477.51)
Dyslipidaemia							
Total	64	43 (67.2%)	-31.32 (-83.33, -14.21)	20 (31.2%)	-10.85 (-46.83, 14.3)	1 (1.6%)	59.48 (59.48, 59.48)
Drug	63	61 (96.8%)	-36.37 (-80.13, -16.09)	2 (3.2%)	0.06 (-8.04, 8.16)	0	-
Non-drug	67	8 (11.9%)	-30.37 (-58.06, -15.49)	49 (73.1%)	3.44 (-115.36, 266.2)	10 (14.9%)	71.43 (39.48, 163.82)
Chronic IHD							
Total	64	20 (31.2%)	-36.57 (-62.03, -10.74)	38 (59.4%)	-5.17 (-82.53, 39.92)	6 (9.4%)	47.82 (25.13, 69.41)
Drug	63	35 (55.6%)	-39.42 (-61.39, -6.32)	24 (38.1%)	-13.39 (-76.32, 80.14)	4 (6.3%)	32.53 (28.06, 67.83)
Non-drug	67	5 (7.5%)	-46.25 (-77.88, -15.91)	42 (62.7%)	0.83 (-638.97, 4382.44)	20 (29.9%)	73.72 (18.79, 403.75)
Diabetes							
Total	54	25 (46.3%)	-26.24 (-50.44, -16.3)	27 (50%)	-11 (-37.81, 20.68)	2 (3.7%)	38.88 (26.64, 51.12)
Drug	54	37 (68.5%)	-26.39 (-58.06, -12.9)	16 (29.6%)	-8.04 (-28.65, 21.73)	1 (1.9%)	29.15 (29.15, 29.15)
Non-drug	59	3 (5.1%)	-18.76 (-66.85, -16)	50 (84.7%)	4.89 (-814.53, 693.88)	6 (10.2%)	92.62 (47.56, 146.18)
**Inpatient Expenditures**							
Chronic IHD							
Total	36	0 (0.0%)	-	33 (91.7%)	0.14 (-8.6, 8.32)	3 (8.3%)	15.66 (6.08, 38.69)
OOP	36	0 (0.0%)	-	31 (86.1%)	2.34 (-5, 27.2)	5 (13.9%)	15.27 (10.16, 47.25)
Drug	36	2 (5.6%)	-18.26 (-23.68, -12.84)	27 (75%)	1.21 (-125.19, 18.27)	7 (19.4%)	14.13 (10.82, 58.98)
Diagnostic	35	10 (28.6%)	-6.62 (-10.54, -2.56)	24 (68.6%)	-2.88 (-7.3, 4.65)	1 (2.9%)	14.31 (14.31, 14.31)
Treat	35	4 (11.4%)	-13.96 (-16.91, -11.83)	31 (88.6%)	-7.46 (-18.2, 0.27)	0	-
Consumable	37	1 (2.7%)	-15.1 (-15.1, -15.1)	33 (89.2%)	7.79 (-374.64, 303.57)	3 (8.1%)	55.78 (14.7, 66.91)
Diabetes							
Total	28	6 (21.4%)	-15.11 (-21.46, -6.71)	20 (71.4%)	-0.27 (-13.5, 14.8)	2 (7.1%)	24.85 (9.84, 39.86)
OOP	31	2 (6.5%)	-15.35 (-17.23, -13.47)	28 (90.3%)	4.8 (-19.16, 13.48)	1 (3.2%)	13.79 (13.79, 13.79)
Drug	29	10 (34.5%)	-31.84 (-55.31, -23.19)	17 (58.6%)	-6.91 (-23.44, 21.75)	2 (6.9%)	20.03 (17.18, 22.87)
Diagnostic	29	0 (0.0%)	-	19 (65.5%)	1.5 (-9.19, 10.25)	10 (34.5%)	14.48 (8.26, 28.67)
Treat	29	9 (31%)	-27.84 (-43.74, -12.83)	16 (55.2%)	2.79 (-27.95, 24.47)	4 (13.8%)	41.04 (21.24, 76.89)
Consumable	31	3 (9.7%)	-24.26 (-29.76, -21.61)	22 (71%)	-1.34 (-113.77, 86.92)	6 (19.4%)	39.99 (17.9, 85.17)

Abbreviations: IHD, ischaemic heart disease; OOP, out-of-pocket. ^a^ The number of hospitals included in each expenditure analysis varied because exclusions for missing values, as well as values below the 5th percentile or above the 95th percentile, were applied separately for each expenditure outcome. For outpatient expenditures, the number of hospitals included in the drug and total expenditure analyses was lower compared to non-drug expenditures. This difference arose from the adjustment for prescription length in the outpatient drug expenditure analysis, with adjustment rates of 88.1% for hypertension, 75.0% for dyslipidaemia, 76.7% for chronic IHD, and 74.0% for diabetes.

####  Inpatient Expenditures

 The relative changes in total expenditures per admission among hospitals ranged from -8.6% to 38.7% for chronic IHD hospitalizations and from -21.5% to 39.9% for diabetes (Table 2). For chronic IHD hospitalizations, no hospitals demonstrated significant decreases in expenditures, with 91.7% (33/36) showing non-significant changes following NVBP implementation. For diabetes, 21.4% (6/28) of hospitals experienced significant decreases in expenditures, while 71.4% (20/28) exhibited non-significant changes (Figure 3, Panel B).

###  Sensitivity Analysis

 Sensitivity analysis results were generally consistent with the main analysis, except for OOP expenditures for diabetes hospitalizations. The sensitivity analysis showed that, compared to the expected expenditures without NVBP, OOP expenditures for diabetes hospitalization increased significantly by 8.48% (95% CI, 5.26% to 12.0%). Detailed sensitivity analysis results are presented in Supplementary file 5.

## Discussion

 To reduce drug expenditure and lower the economic burdens on patients, the Chinese government launched the NVBP program in 2019, which organized national-level centralized drug procurement and promoted the use of lower-priced, centrally procured drugs in public hospitals. While earlier studies have demonstrated the program’s success in reducing drug prices and saving drug procurement expenditures for health systems, its impacts on patients’ medical expenditures remain less well understood. In this study, we used medical records from 1.17 million hypertensive patients across 82 hospitals to investigate the impact of NVBP implementation on medical expenditures for patients. Using interrupted time series analysis, we examined changes in expenditures among hypertensive patient for the treatment of four common medical conditions: hypertension, dyslipidaemia, diabetes, and chronic IHD.

 From an overall perspective, we found the implementation of the NVBP effectively reduced outpatient expenditures among hypertensive patients for the treatment of hypertension, dyslipidaemia, and diabetes by lowering drug expenditures. However, there was no evidence of decreased inpatient expenditures for patients hospitalized for chronic IHD or diabetes. After analyzing the sub-components of expenditures, we found that while drug expenditures for diabetes hospitalization significantly decreased, diagnostic expenditures increased, and there was no significant change in drug expenditures for chronic IHD hospitalization post-NVBP. At the individual hospital level, we found significant variation in policy effects across different hospitals. Despite an overall decrease in outpatient expenditures for hypertension, dyslipidaemia, and diabetes, only 45.6%, 67.2%, and 46.3% of hospitals, respectively, showed significant reductions, while the remainder exhibited either non-significant changes or increases in expenditures.

 We found a significant decrease of 15.61% in total expenditures per visit for patients receiving outpatient care of hypertension following the implementation of the NVBP. Significant reductions were also observed for hypertensive patients managing other related comorbidities, such as dyslipidaemia (-25.77%) and diabetes (-17.59%). These findings highlight the NVBP’s potential to alleviate the economic burden on patients, particularly those with chronic conditions requiring lifelong outpatient prescriptions. The favorable effects of NVBP on outpatient expenditures have also been observed in previous studies that focused on the first pilot round of NVBP. For instance, Li et al documented an 11.4% reduction in total outpatient care expenditures per visit for hypertension patients after NVBP implementation.^34^ Similarly, Lan et al, analyzing encounter data from a single tertiary hospital, observed an immediate reduction of approximately 234 CNY in per-visit drug expenditures for patients treated in outpatient and emergency departments using NVBP bid-winning drugs.^13^

 We did not find significant decreases in total or OOP expenditures per admission for hypertensive patients hospitalized either for diabetes or chronic IHD. However, existing evidence regarding the impact of NVBP on inpatient expenditures has been mixed, with variations observed among different medical condition treatments. Consistent with our study results, Hu et al observed that while diabetes patients who used NVBP drugs during hospitalization had significantly lower drug expenditures compared to those who did not, there was no significant difference in total expenditures between the two patient groups, largely due to the higher diagnostic and consumable expenditures for patients having prescribed lower-priced NVBP drugs.^35^ In contrast, another study analyzing the inpatient expenditures on lung cancer patients found that the implementation of NVBP was associated with significant decreases in total expenditures (-14.13%) and drug expenditures (-20.75%) per inpatient admission, and there were no increases in non-drug-related expenditures.^14^

 By analyzing the sub-components of inpatient expenditures, we found that while drug expenditures for diabetes hospitalization significantly decreased following the NVBP implementation, diagnostic test expenditure increased, resulting in no significant reduction in total expenditures. Similar cost-shifting was observed in the outpatient expenditures for chronic IHD treatment, where drug expenditures decreased significantly, but non-drug-related expenditures increased. These findings align with the theory of physician agency,^10,36^ suggesting that healthcare providers might increase the provision of other high-margin services to offset the potential income loss. Similar patterns have been observed in other pharmaceutical pricing policies, such as the zero-mark-up drug policy.^21^ These findings imply that when considering price cuts for certain services, policy-makers should also monitor potentially unintended impacts on other profitable services.

 Additionally, we observed that drug expenditures for chronic IHD inpatient treatment did not change significantly following NVBP implementation. Since NVBP only covered a subset of commonly used drugs, physicians could still prescribe higher-priced alternative drugs not yet included in the NVBP-drug list to maintain their target income. Previous studies on hospital procurement behavior have also noted that while procurement volumes for lower-priced NVBP-winning drugs increased, undesirable “spillover” effects were observed, with both procurement volumes and expenditures rising for higher-priced alternatives following the implementation of NVBP.^37,38^ However, considering that drug expenditures decreased for treatments of other medical conditions in this study, the non-significant changes observed for chronic IHD might be influenced by demand-side factors. It is possible that the reduction in drug prices resulting from NVBP implementation encouraged patients to purchase more expensive drugs or preventive non-drug services. Further investigation is needed to determine whether the cost-shifting is primarily driven by supply-side or demand-side factors.

 Unlike previous studies that focused exclusively on expenditures for a single medical condition, our study examined both outpatient and inpatient expenditures across multiple conditions within the same patient cohort. This approach enabled a comparison of expenditure changes across different treatments following the implementation of the NVBP program. We found that reductions in drug expenditures varied across medical conditions in outpatient care, with the largest reduction observed in dyslipidaemia (-37.16%), followed by chronic IHD (-25.43%), diabetes (-23.93%), and hypertension (-21.02%). These variations in policy effects may be linked to the differing price reductions for medications covered by the NVBP program (See Supplementary file 1). Specifically, the price reductions for NVBP-covered medications were approximately -73.2% for dyslipidaemia, -60.6% for chronic IHD, -57.9% for diabetes, and -55.6% for hypertension treatment.

 However, from the perspective of the number of medications covered by the NVBP. The NVBP procurement list includes 21 antihypertensive agents, 5 antithrombotic agents, 4 lipid-lowering agents, 2 antianginal agents, and 12 hypoglycemic agents. According to the number of commonly used medications for these conditions listed in clinical guidelines,^39-42^ the NVBP program covered 50.9% (27/53) of medications commonly used for chronic IHD, 30% (12/30) for diabetes, 27% (4/15) for dyslipidaemia, and 27% (21/79) for hypertension. Given this, the NVBP program might have had a larger effect on expenditures for chronic IHD and diabetes. However, the policy’s effects on reducing drug expenditures were also influenced by many other factors, such as the volume of non-NVBP-targeted alternatives prescribed, as well as the proportion of NVBP-covered drugs compared to non-NVBP alternatives used in clinical settings.

 By comparing drug expenditures between outpatient and inpatient treatments, we found that the decline in drug expenditures was less pronounced for inpatient treatment than for outpatient treatment. Additionally, compared to conditions that primarily rely on drug therapies, such as hypertension and dyslipidaemia, conditions requiring more non-drug-related therapies, like chronic IHD and diabetes, showed a more significant increase in non-drug-related expenditures. These findings suggest that treatments requiring more complex therapies were more likely to exhibit smaller reductions in drug expenditures and greater increases in non-drug-related expenditures compared to treatments with simpler therapy strategies. This indicates that future expenditure monitoring could focus more on cases involving relatively complex drug or non-drug therapies.

 Our study is the first study to explore the variation in the effects of the NVBP across individual hospitals. From an overall perspective, we observed significant decreases in outpatient expenditures for the treatment of hypertension, dyslipidaemia, and diabetes after the NVBP. However, at the individual hospital level, only nearly half of the hospitals exhibited significant reductions in treatment expenditures, while the remaining hospitals showed either non-significant changes or increases in expenditures. These findings suggest that the overall decrease in outpatient expenditures was primarily driven by a subset of hospitals that experienced significant reductions. A considerable proportion of hospitals did not show a decrease in expenditures, indicating that there is still potential for further improvement in the overall effectiveness of the policy.

 Further research is needed to understand why some hospitals did not achieve significant reductions in expenditures following the NVBP implementation, and to identify the factors contributed to the variation in policy effects across hospitals. In a previous study assessing the impact of the zero-mark-up drug policy implementation in China, Yip et al found that hospitals more reliant on drug revenue prior to the zero-mark-up policy were more likely to show an increase in expenditures for diagnostic tests and medical consumables, compared to hospitals with less dependence on drug sales.^21^ Such findings suggest that individual hospitals may respond differently to a same policy based on their specific circumstances.

 A previous survey conducted by Zhang et al indicated that different hospitals faced distinct problems and challenges during the implementation of the NVBP policy, and there were variations in the measures adopted to promoting the policy implementation between hospitals, particularly regarding the setting procurement volumes and ensuring the fulfillment of prescribed volumes within the contracted time frame.^43^ These variations in implementation measures may lead to differing policy effects across hospitals. Summarizing the practices and lessons from hospitals with favorable policy effects, and comparing the differences in policy implementation measures between hospitals with favorable and unfavorable policy effects, as identified in this study, might provide valuable insights for improving policy delivery across hospitals in future. This information might be useful for further enhancing the overall effectiveness of policy implementation.

###  Policy Implication

 International evidence suggests that procurement centralization is an effective tool for price reductions by leveraging economies of scale to increase purchasing power.^1^ Successful examples included national and international centralized procurement initiatives for emergency vaccines,^44^ antiviral drugs,^45^ and innovative cancer medications.^5^ An empirical study from Italy suggested that the introduction of the procurement centralization within the regional healthcare systems has effectively reduced per capital health expenditure approximately by 2%-8% for local primary healthcare institutions, underscoring its potential to contain public expenditures for health systems.^12^

 This study provided new evidence on the impact of centralized drug procurement on medical expenditures for patients. Different from centralized procurement experiences in other countries that primarily target high-cost innovative drugs, China’s NVBP program specifically focuses on off-patent drugs with high prescribing volumes in hospitals.^46^ Findings in this study indicate that centralized procurement, combined with measures to ensure the utilization of centrally procured lower-priced drugs in hospitals, might serve as an effective strategy to reduce the economic burden on patients. China’s successful experience may offer valuable lessons for other developing countries facing high pharmaceutical prices and significant patient economic burdens.

 However, given the potential cost-shifting effects from the use of other higher-priced alternative drugs or non-drug services, dynamic monitoring is necessary for other profitable drugs or services, particularly for medical conditions that require more complex drug or non-drug therapies. To mitigate potential cost-shifting and encourage the prescription of low-priced NVBP drugs in clinical settings, the NVBP official document stipulates that savings from its implementation should primarily be allocated to rebalancing the salaries of hospital staff.^17^ How to appropriately use the cost savings from the NVBP to create the right incentives for healthcare providers—guiding their behavior toward more efficient use of cost-effective NVBP drugs and other necessary healthcare services—should be a critical focus for future policy implementation efforts.

 Despite an overall decrease in outpatient expenditures following NVBP implementation, a significant proportion of hospitals showed non-significant changes in expenditures. These findings indicate that while the NVBP can achieve desirable effects, its impact is not uniformly realized across all hospitals. On one hand, these findings highlight the potential for further improvements in policy overall effectiveness. On the other hand, it underscores the need to explore the factors contributing to the variation in policy effects between hospitals, with particular attention to how hospitals implement and respond to the policy in differently.

###  Strength and Limitation 

 This study had two key strengths. First, by using medical records from a cohort of 1.17 million hypertensive patients over five years, we conduct a comprehensive evaluation of the impact of NVBP implementation on medical expenditures among patients for the treatment of multiple common chronic conditions, considering both outpatient and inpatient expenditures, analyzing total expenditures, drug expenditures as well as non-drug expenditures. This is the largest and most comprehensive analysis to date, providing evidence on the evaluation of the NVBP on patient medical expenditures. Second, by applying multilevel modelling technique, we understood not only the general impact of the NVBP program on patient expenditures but also how the policy’s effects vary between individual hospitals. By capturing these nuances, policy-makers can identify specific institutions where the policy is working well and where improvements may be needed.

 There are several limitations. First, our analysis focused solely on the medical expenditures of patients with hypertension, which may limit the generalizability of our findings. To gain a more comprehensive understanding of NVBP’s effectiveness, future research should examine its impact on medical expenditures in other patient subgroups. As NVBP continues to expand and covers a border range of drugs, assessing its impact on medical expenditures across the overall patient population will also be essential. Second, although we have analyzed expenditure changes for four different medical conditions: hypertension, dyslipidaemia, chronic IHD, and diabetes, the expenditure data were derived from a cohort of hypertensive patients. Consequently, the findings regarding expenditure changes for these conditions may only be applicable to hypertensive patients. Third, due to data limitations, we did not explore the factors contributing to variations in policy effects across individual hospitals. Further research is needed to investigate the reasons behind these variations, particularly focusing on how hospitals implement and respond to the policy differently. Finally, the NVBP implementation coincided with the COVID-19 pandemic,^24^ making it challenging to isolate exclusive effect of NVBP. Patients who continued to seek hospital care during the pandemic may have had more severe conditions, potentially introducing an imbalance in clinical characteristics between the pre- and post-NVBP groups. However, if medical expenditures still decreased under this assumption, it would further strengthen our confidence in NVBP’s effectiveness in reducing patient medical expenditures.

## Conclusions

 Based on medical records from a cohort of 1.17 million hypertensive patients, this study found that the implementation of the NVBP in China effectively reduced outpatient expenditures per visit among hypertensive patients for the treatment of hypertension (-15.61%), dyslipidaemia (-25.77%), and diabetes (-17.59%). However, significant increases in non-drug-related expenditures were also observed for several conditions in both outpatient and inpatient care, partially offsetting the overall effectiveness of the policy. This warrants special attention in future policy implementation. Additionally, substantial variation in policy effects were observed across individual hospitals. Despite an overall reduction in outpatient expenditures for hypertension, dyslipidaemia, and diabetes, only 45.6%, 67.2%, and 46.3% of hospitals, respectively, showed significant reductions, while the remaining hospitals exhibited either non-significant changes or increases in expenditures. These findings suggest that there is still room for improvement in policy effectiveness. Future research should explore the factors contributing to the variation in policy effects between hospitals.

## Ethical issues

 Institutional review board approval for this study was obtained from the Medical Ethics Committee of Tianjin WuQing People’s Hospital and the Biomedical Ethics Committee of West China Hospital of Sichuan University. No informed consent was available due to the retrospective design.

## Conflicts of interest

 Authors declare that they have no conflicts of interest.

## Declaimer

 The views expressed in this article are for the authors and not the position of the institutions of affiliation or the funder.

## Data availability statement

 The data that support the findings of this study are available from Tianjin Municipal Health Commission but restrictions apply to the availability of these data, which were used under license for the current study, and so are not publicly available. Data are however available from the authors upon reasonable request and with permission of the Tianjin Municipal Health Commission and the Tianjin Healthcare and Medical Big Data Co., Ltd.

## Supplementary files


Supplementary file 1. Overview of NVBP.

Supplementary file 2. Study Design Consideration.

Supplementary file 3. Analytic Model Specification.

Supplementary file 4. Descriptive Analyses.

Supplementary file 5. Sensitivity Analyses.
