# Supplementary material for: Impact of China’s National Volume-Based Drug Procurement: A Multilevel Interrupted Time Series Analysis on Medical Expenditures in Hypertensive Patients
Source: Int J Health Policy Manag. 2025 May 25;14:8540. doi: 10.34172/ijhpm.8540 (PMC12257203; doi:10.34172/ijhpm.8540)
Supplement: Supplementary file 1 — Overview of NVBP. [file ijhpm-14-8540-s001.pdf]

Article title: Impact of China's National Volume-Based Drug Procurement: A Multilevel Interrupted Time Series Analysis on Medical Expenditures in Hypertensive Patients

Journal name: International Journal of Health Policy and Management (IJHPM)

Authors' information: Yunxiang Huang[1,2,3]¶, Yan Ren[1,2,3]¶, Yuanjin Zhang[1,2,3], Yulong Jia[1,2,3], Qianrui Li[4], Minghong Yao[1,2,3], Yuning Wang[1,2,3], Fan Mei[1,2,3], Kang Zou[1,2,3], Huangang Hu[5], Jing Tan[1,2,3]*, Xin Sun[1,2,3,6]*

[1]Institute of Integrated Traditional Chinese and Western Medicine, and Chinese Evidence-based Medicine Center, West China Hospital, Sichuan University, Chengdu, China.

[2]NMPA Key Laboratory for Real World Data Research and Evaluation in Hainan, Chengdu, China.

[3]Sichuan Center of Technology Innovation for Real World Data, Chengdu, China.

[4]Department of Nuclear Medicine, West China Hospital of Sichuan University, Chengdu, China.

[5]Tianjin Healthcare and Medical Big Data Co., Ltd, Tianjin, China.

[6]Department of Epidemiology and Biostatistics, West China School of Public Health, Sichuan University, Chengdu, China.

***Correspondence to:** Jing Tan; Email: tanjing84@outlook.com Xin Sun; Email: sunxin@wchscu.cn

¶ Both authors contributed equally to this paper.

Citation: Huang Y, Ren Y, Zhang Z, et al. Impact of China's national volume-based drug procurement: a multilevel interrupted time series analysis on medical expenditures in hypertensive patients. *Int J Health Policy Manag.* 2025;14:8540. doi:10.34172/ijhpm.8540

Supplementary file 1. Overview of NVBP

Content 1. Overview of NVBP

Table S1. Timeline for NVBP program

Table S2. Cardiovascular and antidiabetic drugs targeted by the NVBP

Overview of NVBP

Before the implementation of China's National Volume-Based Procurement (NVBP) program, drug procurement was conducted at the provincial level. However, these earlier efforts had limited success in reducing drug prices due to several key issues. First, the bidding process prioritized price but did not link bidding prices to procurement volumes, separating procurement decisions from hospital purchasing quantities. This disconnect made it difficult for pharmaceutical companies to predict sales, reducing their incentive to offer lower prices. Second, the quality of generic drugs—particularly domestically produced ones—varied widely, undermining confidence and limiting competition with off-patent brand-name drugs. As a result, off-patent brands remained significantly more expensive in China compared to other markets. Third, the provincial-level procurement approach constrained economies of scale and reduced overall bargaining power. Finally, there was insufficient coordination among drug quality regulation, pricing strategies, and payment policies. These shortcomings contributed to the limited effectiveness of provincial procurement policies in lowering drug costs.[1]

Under these circumstances, the NVBP program was launched in 2019. It was initially piloted in four municipalities (Beijing, Shanghai, Tianjin, and Chongqing) and seven major cities across different provinces (Guangzhou, Shenzhen, Xi'an, Dalian, Chengdu, Shenyang, and Xiamen), collectively known as the "4+7" procurement reform. Unlike previous provincial-level procurement efforts, the NVBP was implemented at the national level with unprecedented political support. Theoretical analyses suggest that the program has the potential to significantly reduce medical expenditures for patients and improve the sustainability of medical insurance funds by lowering drug prices and encouraging the use of generic medications. The NVBP introduced several key measures that directly impact patients' economic burdens, including pooled national procurement, rigorous quality assurance for generic medicines, and policies that promote the use of generics over branded drugs.

Pooled procurement

The NVBP program seeks to consolidate drug procurement volumes from all public hospitals nationwide to strengthen the bargaining power of hospitals and payers, thereby maximizing price reductions. Participation in the national procurement scheme is mandatory for all public hospitals, which are required to submit projected procurement volumes to the Joint Procurement Office (JPO). Final procurement volumes are determined based on historical purchasing data, clinical usage patterns, market competition, and the number of bidding manufacturers. Typically, the committed procurement volume ranges from 60% to 80% of previous annual volumes. By guaranteeing manufacturers a sizable and predictable market, the NVBP enhances purchasing power and enables the government to negotiate significantly lower prices—effectively implementing a “volume-for-price” strategy.

Quality assurance of generic medicines

To be eligible for inclusion in the NVBP program, products must be approved for marketing in China and listed on the China Marketed Drug Formulary. Eligible products include either originator drugs or generics that have passed the Generic Quality Consistency Evaluation (GQCE). Generics that have not met GQCE standards cannot be included in a therapeutic group if three or more GQCE-approved products already exist within that group. The GQCE ensures that selected generics are safe, effective, and of consistent quality. This creates a competitive environment between generics and off-patent originator drugs. A key innovation of the NVBP reform—unlike previous procurement policies—is its emphasis on direct competition between originator drugs with expired patents and high-quality generics, a strategy designed to further drive down drug prices.

Prompt the use of generic medicines

To ensure that patients can access cost-effective and quality-assured medicines through the national NVBP program, a provincial-level surveillance network has been established to monitor the prescription and utilization of selected drugs. This system ensures that the lower-cost NVBP-selected drugs are more frequently prescribed, dispensed, and used than newer, more expensive alternatives. The use of NVBP-selected drugs in hospitals is included as a performance indicator in annual evaluations, reinforcing institutional accountability. Physicians are required to substitute prescribed medicines with NVBP-selected alternatives when appropriate. In addition, educational campaigns have been launched to raise awareness among healthcare providers about the efficacy

and safety of generic medicines. Preferential co-payment schemes have also been introduced to encourage patients to choose NVBP-listed drugs.

Timeline for NVBP

Until the end of study period in December 2021, Tianjin had carried out five consecutive rounds of NVBP programs. The initial pilot was implemented in April 2019, during which 11 cities including Beijin, Shanghai, and Tianjin grouped together in a tender process to bulk-buy 25 off-patent drugs at more cost-effective rates. Following one year, the second round of NVBP expanded its scope nationwide, involving the participation of all public hospitals across the country. Subsequently, the pace of nationwide drug procurements accelerated, with procurement rounds taking place at increasingly shorter intervals of 6 months, 6 months, and 3 months, respectively **(Table S1)**.

A total of 45 most frequently used cardiovascular medications in clinical practice have been included in the first five rounds of NVBP. Among these, 21 were antihypertensive agents, 12 were hypoglycemic agents, 7 were antithrombotic agents, 4 were lipid-lowering agents, and 3 were antianginal agents **(Table S2)**.

Table S1. Timeline for NVBP program

	4 + 7 pilot	Round 2	Round 3	Round 4	Round 5
Implementation date	Apr 1, 2019	Apr 25, 2020	Nov 1, 2020	May 1, 2021	Sep 1, 2021
Products negotiated [a]	10/25	8/32	7/55	10/44	9/63
Geographic scope	11 cities [b]	National	National	National	National
Procurement contract volume [c]	60% - 70%	1 winner: 50%<br>2 winners: 60%<br>3 winners: 70%<br>≥ 4 winners: 80%	1 winner: 50%<br>2 winners: 60%<br>3 winners: 70%<br>≥ 4 winners: 80%	1 winner: 50%<br>2 winners: 60%<br>3 winners: 70%<br>≥ 4 winners: 80%	1 winner: 50%<br>2 winners: 60%<br>3 winners: 70%<br>≥ 4 winners: 80%
Procurement contract duration	1 year	1 winner: 1 year<br>2-3 winners: 2 years<br>≥ 4 winners: 3 years	1 or 2 winners: 1 year<br>3 winners: 2 years<br>≥ 4 winners: 3 years	1 or 2 winners: 1 year<br>3 winners: 2 years<br>≥ 4 winners: 3 years	1 or 2 winners: 1 year<br>3 winners: 2 years<br>≥ 4 winners: 3 years
Average price reductions	52%	53%	72%	65.4%	56%

[a] The numerator was the cardiovascular and antidiabetic drugs targeted by NVBP, and the denominator was the all products contracted through NVBP; [b] 11 cities included 4 municipalities (Beijing, Tianjin, Shanghai and Chongqing) and 7 major cities from 7 provinces (Chengdu, Xi'an, Shenyang, Dalian, Xiamen, Guangzhou and Shenzhen); [c] Procurement contract volume: the percentage of the purchasing volume compared to the total purchasing volume of last year.

Table S2. Cardiovascular and antidiabetic drugs targeted by NVBP

Timeline	Chemical Substance	ATC code	Chemical Subgroup	Therapeutic Subgroup	Price Change
4+7 Pilot (Apr, 2019)	Irbesartan	C09CA04	ARBs, plain	Agents acting on the renin-angiotensin system	-69.38%
	Losartan Potassium	C09CA01	ARBs, plain	Agents acting on the renin-angiotensin system	-55.13%
	Irbesartan and Hydrochlorothiazide	C09DA04	ARBs, combinations	Agents acting on the renin-angiotensin system	-31.88%
	Fosinopril Sodium	C09AA09	ACE inhibitors, plain	Agents acting on the renin-angiotensin system	-64.13%
	Lisinopril	C09AA03	ACE inhibitors, plain	Agents acting on the renin-angiotensin system	-79.97%
	Enalapril	C09AA02	ACE inhibitors, plain	Agents acting on the renin-angiotensin system	-60.70%
	Amlodipine	C08CA01	Dihydropyridine derivatives	Calcium channel blockers	-57.54%
	Clopidogrel	B01AC04	Platelet aggregation inhibitors	Antithrombotic agents	-63.82%
	Atorvastatin Calcium	C10AA05	HMG CoA reductase inhibitors	Lipid modifying agents	-84.21%
	Rosuvastatin Calcium	C10AA07	HMG CoA reductase inhibitors	Lipid modifying agents	-80.44%
Round 2 (May, 2020)	Olmesartan Medoxomil	C09CA08	ARBs, plain	Agents acting on the renin-angiotensin system	-83.08%
	Candesartan Cilexetil	C09CA06	ARBs, plain	Agents acting on the renin-angiotensin system	-78.46%
	Bisoprolol	C07AB07	Beta blocking agents, selective	Beta blcoking agents	-11.92%
	Indapamide	C03BA11	Sulfonamides, plain	Diuretics	-25.07%

	Simvastatin	C10AA01	HMG CoA reductase inhibitors	Lipid modifying agents	-35.38%
	Terazosin	G04CA03	Alpha-adrenoreceptor antagonists	Urologicals	-24.90%
	Acarbose	A10BF01	Alpha glucosidase inhibitors	Drugs used in diabetes	-78.37%
	Glimepiride	A10BB12	Sulfonylureas	Drugs used in diabetes	-73.23%
	Trimetazidine	C01EB15	Other cardiac preparations	Cardiac therapy	-65.02%
**Round 3 (Nov, 2020)**	Valsartan	C09CA03	ARBs, plain	Agents acting on the renin-angiotensin system	-70.20%
	Captopril	C09AA01	ACE inhibitors, plain	Agents acting on the renin-angiotensin system	-94.71%
	Vildagliptin	A10BH02	DDP-4 inhibitors	Drugs used in diabetes	-76.42%
	Metformin	A10BA02	Biguanides	Drugs used in diabetes	-92.30%
	Apixaban	B01AF02	Direct factor Xa inhibitors	Antithrombotic agents	-96.56%
	Ticagrelor	B01AC24	Platelet aggregation inhibitors	Antithrombotic agents	-82.72%
	Pitavastatin Calcium	C10AA08	HMG CoA reductase inhibitors	Lipid modifying agents	-92.91%
**Round 4 (May, 2021)**	Perindopril Erbumine	C09AA04	ACE inhibitors, plain	Agents acting on the renin-angiotensin system	-76.07%
	Telmisartan	C09CA07	ARBs, plain	Agents acting on the renin-angiotensin system	-45.16%
	Valsartan and Amlodipine	C09DB01	ARBs, combinations	Agents acting on the renin-angiotensin system	-59.25%
	Valsartan and Hydrochlorothiazide	C09DA03	ARBs, combinations	Agents acting on the renin-angiotensin system	-15.63%
	Empagliflozin	A10BK03	SGLT2 inhibitors	Drugs used in diabetes	-56.36%
	Canagliflozin	A10BK02	SGLT2 inhibitors	Drugs used in diabetes	-55.57%
	Repaglinide	A10BX02	Other blood glucose lowering drugs	Drugs used in diabetes	-72.16%
	Nateglinide	A10BX03	Other blood glucose lowering drugs	Drugs used in diabetes	-16.30%
	Gliclazide	A10BB09	Sulfonylureas	Drugs used in diabetes	-58.18%
**Round 5 (Nov, 2021)**	Benazepril	C09AA07	ACE inhibitors, plain	Agents acting on the renin-angiotensin system	-39.15%
	Lercanidipine	C08CA13	Dihydropyridine derivatives	Calcium channel blockers	-41.98%
	Metoprolol	C07AB02	Beta blocking agents, selective	Beta blocking agents	-82.04%
	Miglitol	A10BF02	Alpha glucosidase inhibitors	Drugs used in diabetes	-15.84%
	Saxagliptin	A10BH03	DDP-4 inhibitors	Drugs used in diabetes	-79.12%
	Glipizide	A10BB07	Sulfonylureas	Drugs used in diabetes	-20.79%
	Isosorbide Mononitrate	C01DA14	Organic nitrates	Cardiac therapy	-57.79%
	Dabigatran Etexilate	B01AE07	Direct thrombin inhibitors	Antithrombotic agents	-65.53%
	Rivaroxaban	B01AF01	Direct factor Xa inhibitors	Antithrombotic agents	-98.04%

Drug classifications were referred to the World Health Organization's Anatomical Therapeutic Chemical (ATC) classifications. Price change information was extracted from the website on http://www.yaozh.com; The table does not include non-oral drugs in the NVBP program.

Reference

1. Sunshine Medical Procurement All-In-One. Q&A on the Pilot Program of Centralized Drug Procurement by National Organizations. 2018; https://www.smpaa.cn/gjsdcg/2018/12/08/8534.shtml. Accessed April 15, 2024.
