# Supplementary material for: Impact of China’s National Volume-Based Drug Procurement: A Multilevel Interrupted Time Series Analysis on Medical Expenditures in Hypertensive Patients
Source: Int J Health Policy Manag. 2025 May 25;14:8540. doi: 10.34172/ijhpm.8540 (PMC12257203; doi:10.34172/ijhpm.8540)
Supplement: Supplementary file 2 — Study Design Consideration. [file ijhpm-14-8540-s002.pdf]

Article title: Impact of China's National Volume-Based Drug Procurement: A Multilevel Interrupted Time Series Analysis on Medical Expenditures in Hypertensive Patients

Journal name: International Journal of Health Policy and Management (IJHPM)

Authors' information: Yunxiang Huang[1,2,3]¶, Yan Ren[1,2,3]¶, Yuanjin Zhang[1,2,3], Yulong Jia[1,2,3], Qianrui Li[4], Minghong Yao[1,2,3], Yuning Wang[1,2,3], Fan Mei[1,2,3], Kang Zou[1,2,3], Huangang Hu[5], Jing Tan[1,2,3]*, Xin Sun[1,2,3,6]*

[1]Institute of Integrated Traditional Chinese and Western Medicine, and Chinese Evidence-based Medicine Center, West China Hospital, Sichuan University, Chengdu, China.

[2]NMPA Key Laboratory for Real World Data Research and Evaluation in Hainan, Chengdu, China.

[3]Sichuan Center of Technology Innovation for Real World Data, Chengdu, China.

[4]Department of Nuclear Medicine, West China Hospital of Sichuan University, Chengdu, China.

[5]Tianjin Healthcare and Medical Big Data Co., Ltd, Tianjin, China.

[6]Department of Epidemiology and Biostatistics, West China School of Public Health, Sichuan University, Chengdu, China.

***Correspondence to:** Jing Tan; Email: tanjing84@outlook.com Xin Sun; Email: sunxin@wchscu.cn

¶ Both authors contributed equally to this paper.

Citation: Huang Y, Ren Y, Zhang Z, et al. Impact of China's national volume-based drug procurement: a multilevel interrupted time series analysis on medical expenditures in hypertensive patients. *Int J Health Policy Manag.* 2025;14:8540. doi:10.34172/ijhpm.8540

Supplementary file 2. Study Design Consideration

Content 1. Rationales for using ITS design

Content 2. Events review to validate ITS design

Content 3. ITS impact model selection

Figure S1. NVBP rounds during the study period

Figure S2. Scatter plots of per-visit drug expenditures in outpatient care

Rationales for using ITS design

Interrupted time series (ITS) analysis is a valuable design for evaluating the effects of a population-level intervention.[1-3] Research evidence from ITS design studies could also be included in systematic reviews to inform policy decisions.[4] We considered ITS as an appropriate approach to assess the effectiveness of NVBP program in this study for several reasons. Firstly, there is a clear differentiation of the pre-NVBP period and the post-NVBP period, with well-defined time points for multiple rounds of NVBP as stated in the procurement documents available on the websites of the Sunshine Medical Procurement All-In-One (https://www.smpaa.cn/gjsdcg/index_gg.shtml) and the Tianjin Healthcare Security Administration (https://ylbz.tj.gov.cn/). Secondly, the outcome of interest in this study was expenditure, a short-term outcome that is expected to change immediately after the implementation of NVBP without any delays, so the time point that the NVBP took effect can be pre-specified. Thirdly, we had a large dataset with numerous data points, providing robust support for conducting time series analysis.

Events review to validate ITS design

The validity of ITS analysis might be compromised if any other policy or contextual changes that could influence medical expenditures occurring at the same time as the implementation of the NVBP program. To ensure the accuracy and reliability of our ITS analyses, we systematically reviewed policies and public events that may have affected medical expenditures during the study period. We identified two significant external factors likely to confound the observed changes in expenditures following NVBP implementation: the outbreak of COVID-19 in late December 2019 and the introduction of a long-term prescription policy in February 2020.

The COVID-19 outbreak, which escalated in January 2020, had a profound nationwide impact on healthcare system performance, as documented in a previous study published in *The Lancet Regional Health Western Pacific*.[5] In response, on January 28, 2020, the Tianjin Healthcare Security Administration, in collaboration with the Tianjin Medical Products Administration, implemented a long-term prescription reimbursement policy.[6] This policy allowed qualified physicians to prescribe up to two months of medication for patients with stable chronic conditions—such as hypertension, diabetes, or chronic heart disease—marking a substantial change from the prior two-week prescription standard.

To mitigate the potential confounding effects of these cointerventions, we adopted two methodological adjustments. First, we excluded the four-month period from January to April 2020 from our analysis, as it was marked by significant fluctuations in medical expenditures due to the pandemic. Second, we standardized all drug expenditures for outpatient visits to a 14-day prescription duration to ensure comparability across time.

ITS impact model selection

The propose of impact model was based on both theoretical analysis and data exploration. First, we conducted a theoretical analysis to hypothesize how expenditures would change following the NVBP implementation. We assumed that expenditures would decrease immediately, without any delays, as lower-priced drugs became available to patients shortly after the policy's implementation. As four additional NVBP rounds were carried out consecutively in the years after the pilot phase, the number of lower-priced NVBP drugs gradually increased (**Figure S1**). This gradual expansion may have created a cumulative effect, potentially altering expenditure trends in the post-NVBP period compared to the pre-NVBP period. Furthermore, additional shifts in expenditure levels or trends may have occurred following the implementation of specific NVBP rounds.

To identify the most appropriate impact model that could account for the gradual rollout of NVBP rounds, we conducted an exploratory analysis of per-visit outpatient drug expenditure changes. Outpatient drug expenditures were selected for this analysis because they were the type of expenditure most directly affected by the implementation of the NVBP. Monthly trends of average per-visit outpatient drug expenditures before and after the NVBP implementation were plotted (**Figure S2**). The scatter plot revealed a distinct two-segment change in expenditures following the NVBP implementation, with the second segment occurring at the expansion round of NVBP. This pattern was particularly pronounced in the data on hypertension drug expenditures.

Based on both theorical analysis and data exploration, we finally chose a two-intervention ITS model. This process of determining the impact model aligns with the recommendations provided in existing ITS analysis tutorials.[1]

On the other hand, although the study period (2017-2021) covers five rounds of NVBP, a five-segment regression model might not be the optimal choice. A five-segment regression approach

would require estimating 12 parameters (1 intercept, 1 trend before NVBP, and 5 pairs of level and trend changes for each round of NVBP implementation) using only 60 time points in the time series. While technically feasible, the limited number of data points before and after each round of NVBP would likely lead to unreliable estimates.[1,7]

Most importantly, the primary objective of this study was not to examine whether there were significant level or trend changes in expenditures at discrete points following each NVBP round. Instead, we evaluated the NVBP effect from a broader perspective, treating all rounds of NVBP as a whole rather than assessing each round individually. To better interpret the overall effect of NVBP, we calculated a relative change indicator, combining level and trend changes into a single estimate.[8] The relative changes in expenditures were calculated by comparing model-predicted expenditures with and without the interventions. In this context, the key methodological consideration was selecting an appropriate model that captures the actual levels and trends in expenditures following the NVBP implementation. This is also why we combined theoretical and exploratory analysis to determine the impact model.

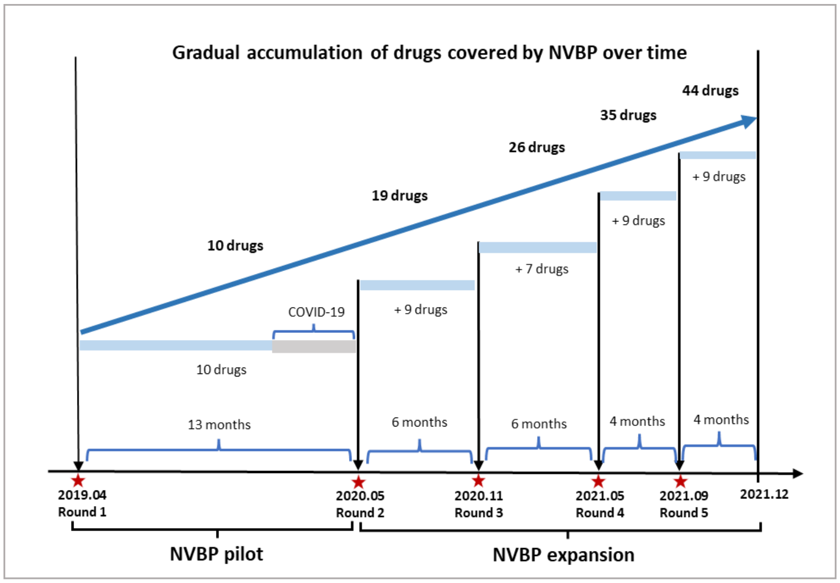

Figure S1. NVBP Rounds During the Study Period. The figure shows the number of cardiovascular and anti-diabetes drugs included in the program.

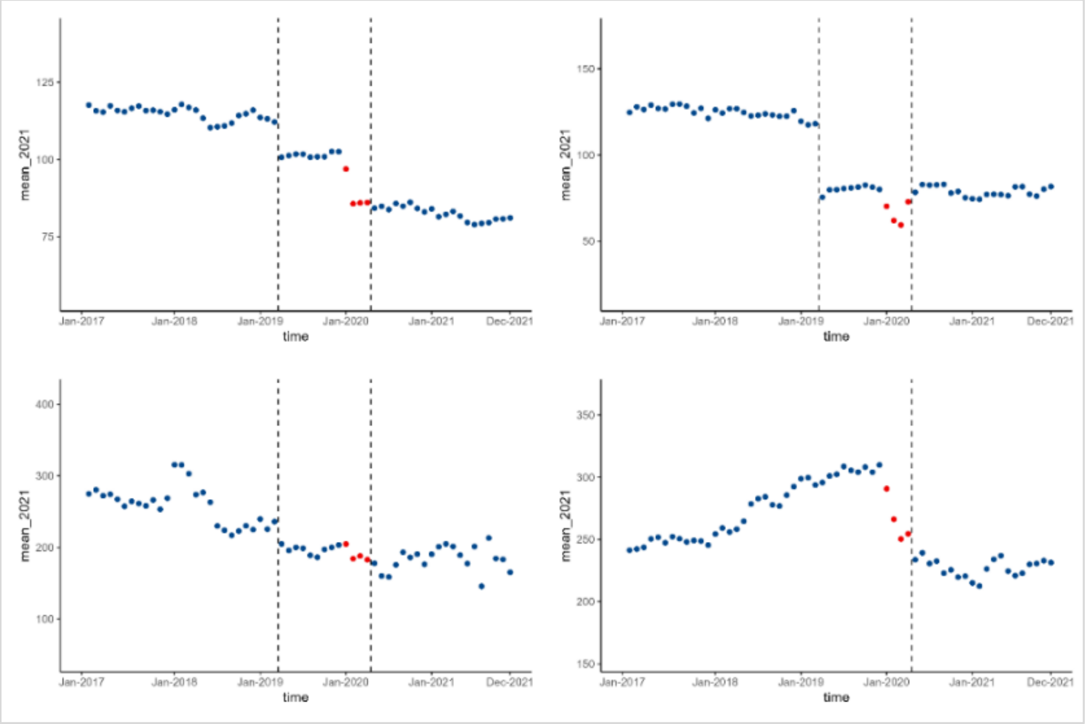

Figure S2. Scatter Plots of Per-Visit Drug Expenditures in Outpatient Care. Points represent the monthly average drug expenditures per medical visit before and after the NVBP. Red points indicate the mean monthly drug expenditure per visit during the COVID-19 outbreak period (January to April 2020).

Reference

1. Bernal JL, Cummins S, Gasparrini A. Interrupted time series regression for the evaluation of public health interventions: a tutorial. *Int J Epidemiol.* 2017;46(1):348-355. doi:10.1093/ije/dyw098

2. Bhaskaran K, Gasparrini A, Hajat S, Smeeth L, Armstrong B. Time series regression studies in environmental epidemiology. *Int J Epidemiol.* 2013;42(4):1187-1195. doi:10.1093/ije/dyt092

3. Kontopantelis E, Doran T, Springate DA, Buchan I, Reeves D. Regression based quasi-experimental approach when randomisation is not an option: interrupted time series analysis. *Bmj.* 2015;350:h2750. doi:10.1136/bmj.h2750

4. Acosta A, Ciapponi A, Aaserud M, et al. Pharmaceutical policies: effects of reference pricing, other pricing, and purchasing policies. *Cochrane Database Syst Rev.* 2014;2014(10):Cd005979. doi:10.1002/14651858.CD005979.pub2

5. Xiao H, Dai X, Wagenaar BH, et al. The impact of the COVID-19 pandemic on health services utilization in China: Time-series analyses for 2016-2020. *Lancet Reg Health West Pac.* 2021;9:100122. doi:10.1016/j.lanwpc.2021.100122

6. Tianjin Healthcare Security Administration. Implementing Long-term Prescriptions to Assist in Epidemic Prevention and Control. 2020; https://ylbz.tj.gov.cn/xwzx/ylbzxx/202009/t20200916_3701440.html. Accessed April 15, 2024.

7. Wagner AK, Soumerai SB, Zhang F, Ross-Degnan D. Segmented regression analysis of interrupted time series studies in medication use research. *J Clin Pharm Ther.* 2002;27(4):299-309. doi:10.1046/j.1365-2710.2002.00430.x

8. Zhang F, Wagner AK, Soumerai SB, Ross-Degnan D. Methods for estimating confidence intervals in interrupted time series analyses of health interventions. *J Clin Epidemiol.* 2009;62(2):143-148. doi:10.1016/j.jclinepi.2008.08.007
