# Supplementary material for: Impact of China’s National Volume-Based Drug Procurement: A Multilevel Interrupted Time Series Analysis on Medical Expenditures in Hypertensive Patients
Source: Int J Health Policy Manag. 2025 May 25;14:8540. doi: 10.34172/ijhpm.8540 (PMC12257203; doi:10.34172/ijhpm.8540)
Supplement: Supplementary file 3 — Analytic Model Specification. [file ijhpm-14-8540-s003.pdf]

Article title: Impact of China's National Volume-Based Drug Procurement: A Multilevel Interrupted Time Series Analysis on Medical Expenditures in Hypertensive Patients

Journal name: International Journal of Health Policy and Management (IJHPM)

Authors' information: Yunxiang Huang[1,2,3]¶, Yan Ren[1,2,3]¶, Yuanjin Zhang[1,2,3], Yulong Jia[1,2,3], Qianrui Li[4], Minghong Yao[1,2,3], Yuning Wang[1,2,3], Fan Mei[1,2,3], Kang Zou[1,2,3], Huangang Hu[5], Jing Tan[1,2,3]*, Xin Sun[1,2,3,6]*

[1]Institute of Integrated Traditional Chinese and Western Medicine, and Chinese Evidence-based Medicine Center, West China Hospital, Sichuan University, Chengdu, China.

[2]NMPA Key Laboratory for Real World Data Research and Evaluation in Hainan, Chengdu, China.

[3]Sichuan Center of Technology Innovation for Real World Data, Chengdu, China.

[4]Department of Nuclear Medicine, West China Hospital of Sichuan University, Chengdu, China.

[5]Tianjin Healthcare and Medical Big Data Co., Ltd, Tianjin, China.

[6]Department of Epidemiology and Biostatistics, West China School of Public Health, Sichuan University, Chengdu, China.

***Correspondence to:** Jing Tan; Email: tanjing84@outlook.com Xin Sun; Email: sunxin@wchscu.cn

¶ Both authors contributed equally to this paper.

Citation: Huang Y, Ren Y, Zhang Z, et al. Impact of China's national volume-based drug procurement: a multilevel interrupted time series analysis on medical expenditures in hypertensive patients. *Int J Health Policy Manag.* 2025;14:8540. doi:10.34172/ijhpm.8540

Supplementary file 3. Analytic Model Specification

Content 1. Two-level regression model equation

Content 2. Relative change estimation equation

Two-level regression model equation

$$y_{ij} = \beta_0 + \beta_1 time_{ij} + \beta_2 pilot_{ij} + \beta_3 time\_after\_pilot_{ij} + \beta_4 expansion_{ij} + \beta_5 time\_after\_expansion_{ij} + \beta_6 X_{ij} + u_{0j} + u_{2j} pilot_{ij} + u_{4j} expansion_{ij} + \varepsilon_{ii}$$

$$\beta_{0j} = \beta_0 + u_{0j}$$

$$\beta_{2j} = \beta_2 + u_{2j}$$

$$\beta_{4j} = \beta_4 + u_{4j}$$

In this regression equation, y_{ij} is the monthly mean expenditures per patient visit for month i in hospital j. $time$ is the month number from 1 to 56 where 1= January 2017, 56 = December 2021 (months from January 2020 to April 2020 were excluded from model due to the COVID-19 outbreak). $pilot$ is the binary indicator for NVBP pilot from April 2019 to December 2021 equal to 1 if the month occurred in that period and 0 otherwise. *time_after_pilot* is the trend count variable for the NVBP pilot period where 0 = before April 2019, 1 = April 2019, and 29 = December 2021. *expansion* is the binary indicator for NVBP expansion from May, 2020 to December 31, 2021 equal to 1 if the month occurred in that period and 0 otherwise. *time_after_expansion* is the trend count variable for the NVBP expansion period where 0 = before May 2020, 1 = May 2020, and 20 = December 2021.

The β_0, β_1, β_2, β_3, β_4, β_5, β_6 in equation are regression coefficients, representing the overall average effects across all hospitals. β_0 is the intercept. β_1 is the trend in expenditures before the NVBP pilot program. β_2 is the immediate level change in expenditures following the NVBP pilot. $\beta_3$is the change in the trend of expenditures during the NVBP pilot period, compared to the preNVBP pilot period. β_4 is the immediate level change in expenditures following the NVBP expansion, while β_5 is the trend change in expenditures during the NVBP expansion period, relative to the trend during the NVBP pilot period. β_6 is a vector of estimates corresponding to the individual covariates, which is the monthly proportion of male patients, the mean age of patients, the mean number of unique medications prescribed, comorbid diagnoses (in inpatient expenditure models only), and dummy variables for calendar months.

The u_{0j}, u_{2j}, u_{4j} in equation are random residual error terms at the hospital level, accounting for the hospital-specific variance of the regression coefficients for β_0, β_2, and β_4 respectively. By incorporating u_{0j}, u_{2j}, u_{4j}, we estimated the different coefficients of intercept (β_{0j}), as well as the different coefficients of level changes in expenditures post-NVBP pilot (β_{2j}) and NVBP expansion (β_{4j}) across each hospital.

The random error terms for two policy effect parameters—the trend changes in expenditures postNVBP pilot (u_{3j}) and NVBP expansion (u_{5j})—were not included in the two-level regression

model. This decision was based on the small variance of the residual errors, which indicated minimal variation in the trend changes in expenditures between hospitals after the NVBP pilot and expansion. Additionally, including these variance components led to convergence issues in the model,[1] further supporting their exclusion. To balance type I error and statistical power,[2] the outpatient expenditure models included intercept, as well as both level changes associated with the NVBP pilot and NVBP expansion as random error terms at the hospital level. In contrast, for the inpatient expenditure models, only intercept and the level change for the NVBP expansion were included as random error terms at the hospital level.

Relative Changes Estimation

$$Relative\ change = 100 * \sum \underline{\qquad\qquad\qquad\qquad} {}^{T}{}_{t=t_*+1}(\hat{Y}\ _{t}\sum{}_{witT\ h}) - (\hat{Y}\sum{}_{t}$$

$$_{wit}{}^{T}{}_{t=th_*+out1}()\hat{Y}\ _{t\ without}) \quad t \in \{t_*, \dots, T)$$

$$t=t_*+1$$

Where t^* is the first month after the NVBP implementation (April 2019). T is the last month after the NVBP implementation observed in this study (December 2021). t is the month for which expected expenditures were calculated under the ITS model. In this study, we first calculated the predicted expenditures for each month from April 2019 to December 2021. $\sum^{T}{}_{t=t_*+1}(\hat{Y}_{t\ with})$ is the average predicted expenditures over the entire post-NVBP period (factual scenario).

$\sum^{T}{}_{t=t_*+1}(\hat{Y}_{t\ without})$ is the average predicted expenditures over the entire post-NVBP period assuming that the NVBP had not been implemented (counterfactual scenario).

Reference

1. Brown VA. An Introduction to Linear Mixed-Effects Modeling in R. *Advances in Methods and Practices in Psychological Science.* 2021;4(1):2515245920960351. doi:10.1177/2515245920960351

2. Matuschek H, Kliegl R, Vasishth S, Baayen H, Bates D. Balancing Type I error and power in linear mixed models. *Journal of Memory and Language.* 2017;94:305-315. doi:10.1016/j.jml.2017.01.001
