# Supplementary material for: Impact of China’s National Volume-Based Drug Procurement: A Multilevel Interrupted Time Series Analysis on Medical Expenditures in Hypertensive Patients
Source: Int J Health Policy Manag. 2025 May 25;14:8540. doi: 10.34172/ijhpm.8540 (PMC12257203; doi:10.34172/ijhpm.8540)
Supplement: Supplementary file 4 — Descriptive Analyses. [file ijhpm-14-8540-s004.pdf]

Article title: Impact of China's National Volume-Based Drug Procurement: A Multilevel Interrupted Time Series Analysis on Medical Expenditures in Hypertensive Patients

Journal name: International Journal of Health Policy and Management (IJHPM)

Authors' information: Yunxiang Huang[1,2,3]¶, Yan Ren[1,2,3]¶, Yuanjin Zhang[1,2,3], Yulong Jia[1,2,3], Qianrui Li[4], Minghong Yao[1,2,3], Yuning Wang[1,2,3], Fan Mei[1,2,3], Kang Zou[1,2,3], Huangang Hu[5], Jing Tan[1,2,3]*, Xin Sun[1,2,3,6]*

[1]Institute of Integrated Traditional Chinese and Western Medicine, and Chinese Evidence-based Medicine Center, West China Hospital, Sichuan University, Chengdu, China.

[2]NMPA Key Laboratory for Real World Data Research and Evaluation in Hainan, Chengdu, China.

[3]Sichuan Center of Technology Innovation for Real World Data, Chengdu, China.

[4]Department of Nuclear Medicine, West China Hospital of Sichuan University, Chengdu, China.

[5]Tianjin Healthcare and Medical Big Data Co., Ltd, Tianjin, China.

[6]Department of Epidemiology and Biostatistics, West China School of Public Health, Sichuan University, Chengdu, China.

***Correspondence to:** Jing Tan; Email: tanjing84@outlook.com Xin Sun; Email: sunxin@wchscu.cn

¶ Both authors contributed equally to this paper.

Citation: Huang Y, Ren Y, Zhang Z, et al. Impact of China's national volume-based drug procurement: a multilevel interrupted time series analysis on medical expenditures in hypertensive patients. *Int J Health Policy Manag.* 2025;14:8540. doi:10.34172/ijhpm.8540

Supplementary file 4. Descriptive Analyses

Figure S1. Study population flowchart

Table S1. Study cohort characteristics

Table S2. Count of medical visits for different conditions pre- and post-NVBP

Table S3. Summary statistics of outpatient expenditures for different conditions pre- and postNVBP.

Table S4. Summary statistics of inpatient expenditures for different conditions pre- and post-NVBP

Figure S1. Study population flowchart

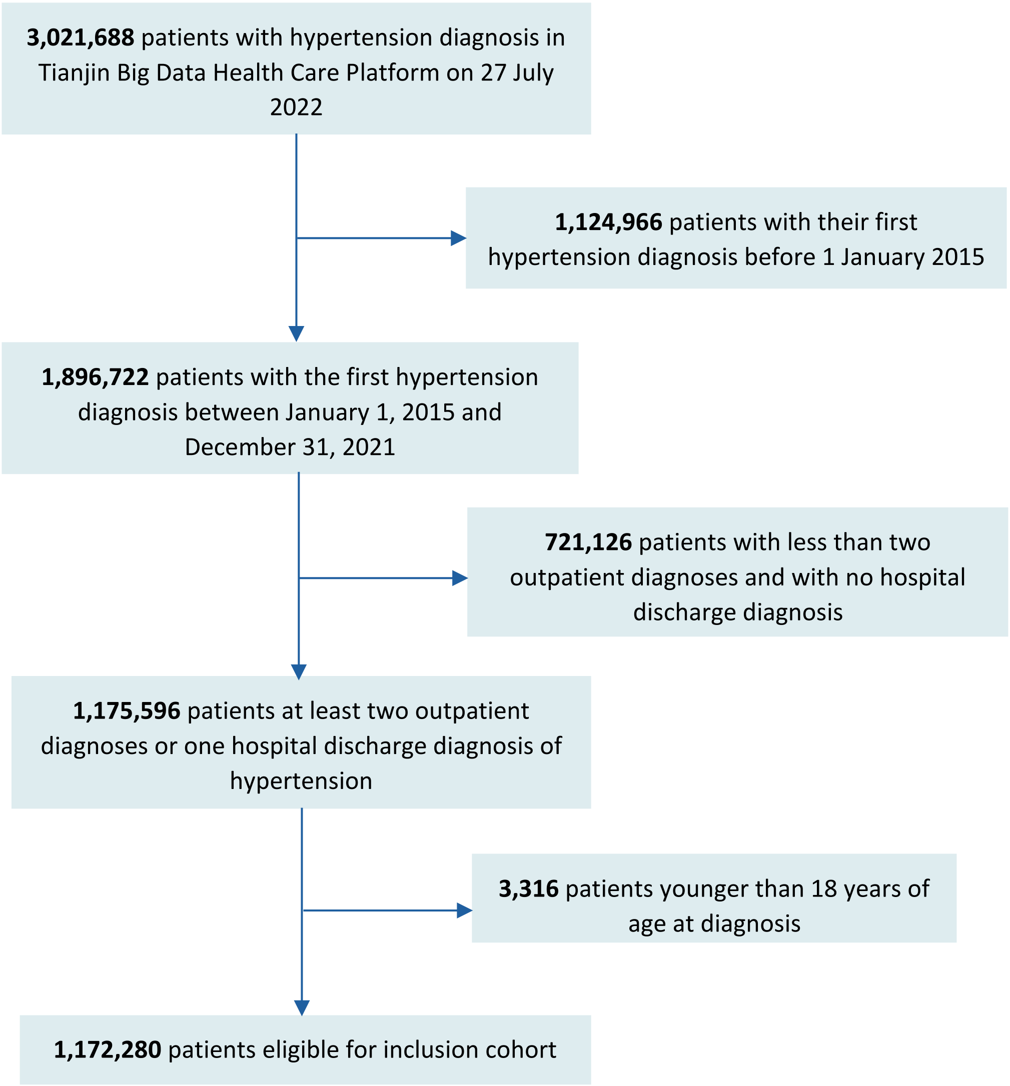

Table S1. Study cohort characteristics

Characteristics	Overall Count (%)	Pre-NVBP Count (%)	Post-NVBP Count (%)
Patients	1,172,280	873,528	108,0815
Year of diagnosis			
2015	229,214 (19.55)	229,214 (26.24)	200,123 (18.52)
2016	217,319 (18.54)	217,319 (24.88)	192,998 (17.86)
2017	208,730 (17.81)	208,730 (23.9)	187,066 (17.31)
2018	174,043 (14.85)	174,043 (19.92)	159,748 (14.78)
2019	155,569 (13.27)	44,222 (5.06)	153,475 (14.2)
2020	107,285 (9.15)	-	107,285 (9.93)

2021	80,120 (6.83)	-	80,120 (7.41)
Sex			
Male	607,819 (51.85)	449,889 (51.5)	557,468 (51.58)
Female	564,461 (48.15)	423,639 (48.5)	523,347 (48.42)
Age, median (IQR)	58 (47-66)	58 (48-66)	57 (47-66)
Age group			
18-25	109,98 (0.94)	6,931 (0.81)	10,079 (0.93)
26-30	34,364 (2.93)	25,516 (2.98)	32,086 (2.97)
31-35	59,352 (5.06)	42,822 (5.01)	56,171 (5.2)
36-40	70,648 (6.03)	49,130 (5.74)	67,010 (6.2)
41-45	80,045 (6.83)	58,981 (6.89)	75,582 (6.99)
46-50	105,539 (9)	76,641 (8.96)	99,476 (9.2)
51-55	150,593 (12.85)	115,607 (13.51)	140,356 (12.99)
56-60	170,638 (14.56)	126,358 (14.77)	159,369 (14.75)
61-65	180,756 (15.42)	132,967 (15.54)	167,749 (15.52)
66-70	129,561 (11.05)	90,164 (10.54)	119,048 (11.01)
71-75	81,766 (6.97)	58,271 (6.81)	73,522 (6.8)
> 75	98,020 (8.36)	72,137 (8.43)	80,363 (7.44)
Cardiovascular history [a]			
Chronic ischaemic heart disease	385,726 (32.9)	307,862 (35.24)	351,014 (32.48)
Cerebrovascular disease	282,638 (24.11)	208,277 (23.84)	255,379 (23.63)
Atrial fibrillation	109,554 (9.35)	80,437 (9.21)	99,564 (9.21)
Angina pectoris	73,421 (6.26)	54,261 (6.21)	67,533 (6.25)
Heart failure	69,784 (5.95)	53,067 (6.08)	60,254 (5.57)
Atherosclerosis	51,045 (4.35)	37,220 (4.26)	47,010 (4.35)
Acute ischaemic heart disease	40,610 (3.46)	26,761 (3.06)	38,196 (3.53)
Myocardial infarction	20,189 (1.72)	13,889 (1.59)	18,142 (1.68)
General medical history [1]			
Dyslipidemia	265,399 (22.64)	196,897 (22.54)	248,126 (22.96)
Diabetes	201,303 (17.17)	150,554 (17.24)	186,334 (17.24)
Skin viral infections	168,434 (14.37)	111,334 (12.75)	160,852 (14.88)
Acute upper respiratory infections	155,485 (13.26)	122,509 (14.02)	145,659 (13.48)
Dizziness and giddiness	150,347 (12.83)	97,834 (11.2)	142,209 (13.16)
Dorsopathies	138,167 (11.79)	101,823 (11.66)	131,232 (12.14)
Gastritis and duodenitis	135,378 (11.55)	100,979 (11.56)	127,539 (11.8)
Intestinal infectious diseases	132,531 (11.31)	90,002 (10.3)	125,826 (11.64)
Respiratory symptoms	119,679 (10.21)	81,754 (9.36)	113,511 (10.5)
Arthropathies	100,217 (8.55)	73,763 (8.44)	95,438 (8.83)
Metabolic disorders	95,336 (8.13)	63,856 (7.31)	85,240 (7.89)
Chronic lower respiratory disease	89,941 (7.67)	69,543 (7.96)	83,224 (7.7)
Liver disease	82,937 (7.07)	60,635 (6.94)	76,931 (7.12)
Digestive and abdominal symptoms	82,010 (7)	55,726 (6.38)	77,983 (7.22)
Sleep disorders	81,047 (6.91)	57,415 (6.57)	76,913 (7.12)
Dermatitis and eczema	52,755 (4.5)	37,028 (4.24)	50,854 (4.71)
Headache	50,033 (4.27)	31,372 (3.59)	47,967 (4.44)
Medication use [a]			
Anti-hypertensive drugs [b]	865,158 (73.8)	652,151 (74.66)	796,353 (73.68)
Calcium channel blockers	478,157 (40.79)	362,911 (41.55)	437,808 (40.51)
ARB blockers	287,792 (24.55)	220,896 (25.29)	266,868 (24.69)
Beta-blockers	228,683 (19.51)	171,961 (19.69)	209,215 (19.36)
Fixed-dose combination	128,154 (10.93)	89,915 (10.29)	118,652 (10.98)

ACEI	99,790 (8.51)	83,425 (9.55)	88,569 (8.19)
Diuretics	72,588 (6.19)	53,967 (6.18)	60,657 (5.61)
Lipid-lowering agents	325,224 (27.74)	244,825 (28.03)	297,148 (27.49)
Antithrombotic agents	321,547 (27.43)	247,748 (28.36)	291,602 (26.98)
Antianginal agents	195,102 (16.64)	154,037 (17.63)	173,596 (16.06)
Oral hypoglycemic agents	133,368 (11.38)	100,558 (11.51)	124,449 (11.51)

ACEI = angiotensin-converting enzyme inhibitors, ARB = angiotensin receptor blockers. [a] Patient was the unite of descriptive analysis, the numerator was the number of patients with according medical diagnoses 12 month before the index data (the time of first being diagnosed with hypertension in database), and the dominator was the number of patients in responding time period. [b] Anti-hypertensive agents included diuretics, calcium channel blockers, angiotensin-converting enzyme inhibitors, angiotensin receptor blockers, renin-inhibitors, beta-blockers, alphaadrenoreceptor antagonists, centrally active drugs, arteriolar smooth muscle, traditional compound drugs and fixed-dose combination drugs.

Table S2. Count of medical visits for different conditions pre- and post-NVBP.

**Medical visits**	**Overall**	**Pre-NVBP**	**Post-NVBP**
	Jan 2017 – Dec 2021<br>Count (%)	Jan 2017 – Mar 2019<br>Count (%)	Apr 2019 – Dec 2021<br>Count (%)
Medical visits			
Outpatient visits	3,225,890	1,680,124	1,545,766
Inpatient admissions	68,491	42,020	26,471
Outpatient visits by condition			
Hypertension	1,827,617	883,632	943,985
Dyslipidemia	234,076	100,761	133,315
Chronic IHD	475,736	247,033	228,703
Diabetes *	688,461	448,698	239,763
Inpatient admissions by condition			
Chronic IHD	28,729	14,395	14,334
Diabetes *	39,762	27,625	12,137

* The analysis of diabetes data prior to the NVBP extended from January 2017 to April 2020; Chronic IHD = chronic ischaemic heart disease.

Table S3. Summary statistics of outpatient expenditures for different conditions pre- and post-NVBP.

	**Pre-NVBP**<br>Jan 2017 – Mar 2019<br>Mean (SD)	**Post-NVBP**<br>Apr 2019 – Dec 2021<br>Mean (SD)
**Hypertension**		
Total	145.89 (5.23)	116.65 (7.58)
Drug	114.83 (2.17)	88.37 (9.12)
Non-drug	17.37 (1.09)	21.41 (3.36)
**Dyslipidemia**		
Total	159.47 (5.45)	119.29 (6.81)
Drug	124.79 (3.22)	79.3 (2.72)
Non-drug	36.83 (3.88)	44.37 (5.66)
**Chronic IHD**		
Total	357.11 (24.52)	339.43 (40.93)
Drug	259.13 (27.33)	187.99 (15.87)
Non-drug	61.27 (8.27)	101.55 (33.35)
**Diabetes**		
Total	352.92 (25.64)	337.52 (12.86)
Drug	274.22 (24.26)	227.12 (7.23)
Non-drug	46.44 (3.53)	60.85 (4.2)

The analysis of diabetes data prior to the NVBP extended from January 2017 to April 2020; Chronic IHD = chronic ischaemic heart disease.

Table S4. Summary statistics of inpatient expenditures for different conditions pre- and post-NVBP

	**Pre-NVBP**<br>Jan 2017 – Mar 2019<br>Mean (SD)	**Post-NVBP**<br>Apr 2019 – Dec 2021<br>Mean (SD)
**Chronic IHD**		
Total	12569.01 (498.07)	12114.46 (551.39)
OOP	4735.14 (228.73)	4499.29 (236.91)
Drug	2674.43 (189.8)	2199.13 (293.42)
Diagnostic	3620.78 (93.59)	3640.93 (261.61)
Treat	2654.13 (162.46)	2663.4 (175.05)
Consumable	754.37 (67.02)	963.74 (156.44)
**Diabetes**		
Total	10501.61 (267.85)	10603.68 (409.88)
OOP	3363.54 (176.75)	3431.24 (132.27)
Drug	2752.84 (171.41)	2228.29 (112.6)
Diagnostic	4065.46 (67.03)	4443.33 (192.19)
Treat	1429.81 (72.83)	1284.41 (97.19)
Consumable	505.38 (75.37)	514.19 (28.36)

The analysis of diabetes data prior to the NVBP extended from January 2017 to April 2020; Chronic IHD = chronic ischaemic heart disease; OOP=out-of-pocket.
