# Supplementary material for: Impact of China’s National Volume-Based Drug Procurement: A Multilevel Interrupted Time Series Analysis on Medical Expenditures in Hypertensive Patients
Source: Int J Health Policy Manag. 2025 May 25;14:8540. doi: 10.34172/ijhpm.8540 (PMC12257203; doi:10.34172/ijhpm.8540)
Supplement: Supplementary file 5 — Sensitivity Analyses. [file ijhpm-14-8540-s005.pdf]

Article title: Impact of China's National Volume-Based Drug Procurement: A Multilevel Interrupted Time Series Analysis on Medical Expenditures in Hypertensive Patients
Journal name: International Journal of Health Policy and Management (IJHPM)
Authors' information: Yunxiang Huang[1,2,3]¶, Yan Ren[1,2,3]¶, Yuanjin Zhang[1,2,3], Yulong Jia[1,2,3], Qianrui Li[4], Minghong Yao[1,2,3], Yuning Wang[1,2,3], Fan Mei[1,2,3], Kang Zou[1,2,3], Huangang Hu[5], Jing Tan[1,2,3]*, Xin Sun[1,2,3,6]*

[1]Institute of Integrated Traditional Chinese and Western Medicine, and Chinese Evidence-based Medicine Center, West China Hospital, Sichuan University, Chengdu, China.

[2]NMPA Key Laboratory for Real World Data Research and Evaluation in Hainan, Chengdu, China.

[3]Sichuan Center of Technology Innovation for Real World Data, Chengdu, China.

[4]Department of Nuclear Medicine, West China Hospital of Sichuan University, Chengdu, China.

[5]Tianjin Healthcare and Medical Big Data Co., Ltd, Tianjin, China.

[6]Department of Epidemiology and Biostatistics, West China School of Public Health, Sichuan University, Chengdu, China.

***Correspondence to:** Jing Tan; Email: tanjing84@outlook.com Xin Sun; Email: sunxin@wchscu.cn

¶ Both authors contributed equally to this paper.

Citation: Huang Y, Ren Y, Zhang Z, et al. Impact of China's national volume-based drug procurement: a multilevel interrupted time series analysis on medical expenditures in hypertensive patients. *Int J Health Policy Manag.* 2025;14:8540. doi:10.34172/ijhpm.8540

Supplementary file 5. Sensitivity Analyses

Table S1. Segmented generalized least squares models on outpatient expenditures

Table S2. Segmented generalized least squares models on inpatient expenditures

Figure S1. Interrupted time series plots of outpatient expenditures

Figure S2. Interrupted time series plots of inpatient expenditures

Figure S3. Relative changes (%) in outpatient expenditures after NVBP

Figure S4. Relative changes (%) in inpatient expenditures after NVBP

Table S1. Segmented generalized least squares models on outpatient expenditures

Outpatient expenditures	Intercept	Trend before NVBP	NVBP pilot (April 2019-January 2020)		NVBP expansion (May 2020 – December 2021)	
			Level change	Slope change	Level change	Slope change
	β_0 (95% CI)	β_1 (95% CI)	B_2 (95% CI)	B_3 (95% CI)	B_4 (95% CI)	B_5 (95% CI)
**Hypertension**						
Total	151.41 (148.21 to 154.6)	-0.46 (-0.69 to -0.22) ***	-12.98 (-16.27 to -9.69) ***	0.49 (-0.12 to 1.09)	-7.04 (-11.94 to -2.13) **	-0.31 (-0.86 to 0.24)
Drug	117.06 (115.02 to 119.11)	-0.14 (-0.29 to 0.01)	-13.2 (-15.27 to -11.13) ***	0.23 (-0.15 to 0.61)	-12.46 (-15.27 to -9.64) ***	-0.47 (-0.82 to -0.11) **
Non-drug	17.17 (15.5 to 18.83)	-0.05 (-0.13 to 0.03)	0.03 (-1.23 to 1.29)	0.09 (-0.11 to 0.28)	7.52 (5.43 to 9.62) ***	0.03 (-0.16 to 0.22)
**Dyslipidemia**						
Total	154.46 (145.86 to 163.05)	-0.05 (-0.51 to 0.4)	-44.71 (-52.23 to -37.18) ***	1.56 (0.33 to 2.79) *	4.41 (-3.2 to 12.01)	-1.24 (-2.63 to 0.16)
Drug	121.03 (115.9 to 126.15)	-0.03 (-0.28 to 0.22)	-44.61 (-49.45 to -39.76) ***	0.91 (0.12 to 1.71) *	-4.07 (-8.8 to 0.65)	-1.01 (-1.88 to -0.15) *
Non-drug	28.75 (21.47 to 36.02)	0.25 (-0.1 to 0.61)	-0.74 (-6.3 to 4.81)	0.01 (-0.92 to 0.93)	9.56 (3.45 to 15.68) **	-0.16 (-1.17 to 0.85)
**Chronic IHD**						
Total	328.54 (297.12 to 359.96	0.21 (-3.39 to 3.8)	-18.77 (-54.93 to 17.4)	-2.14 (-10.21 to 5.93)	103.39 (61.39 to 145.4) ***	-0.17 (-8.12 to 7.79)
Drug	248.23 (217.19 to 279.26)	-0.09 (-1.73 to 1.54)	-30.25 (-60.09 to -0.41) *	-1.54 (-6.87 to 3.8)	14.62 (-17.55 to 46.8)	1.5 (-3.72 to 6.72)
Non-drug	66.87 (45.56 to 88.18)	0.71 (-0.36 to 1.79)	-4.46 (-21.94 to 13.03)	1.83 (-1.86 to 5.53)	54.85 (34.11 to 75.6) ***	-3.55 (-7.22 to 0.12)
**Diabetes**						
Total	322.56 (304.61 to 340.51)	1.68 (0.74 to 2.61) ***	-	-	-60.04 (-80.18 to -39.9) ***	-0.7 (-2.22 to 0.82)
Drug	250.49 (235.96 to 265.02)	1.16 (0.52 to 1.8) ***	-	-	-71.47 (-82.42 to -60.52) ***	-0.98 (-2.37 to 0.41)
Non-drug	47.28 (41.63 to 52.93)	-0.12 (-0.41 to 0.17)	-	-	11.23 (5.25 to 17.21) ***	0.43 (0.01 to 0.85) *

The analysis of diabetes before the implementation of NVBP spanned from January 2017 to April 2020; Non-drugs were calculated as the total expenditures of outpatient care minus drug expenditures; Chronic IHD = chronic ischaemic heart disease; *** p < 0.001; ** p<0.01; * p<0.05;

Table S2. Segmented generalized least squares models on inpatient expenditures

Inpatient expenditures	Intercept β_0 (95% CI)	Trend before NVBP β_1 (95% CI)	NVBP pilot (April 2019-January 2020)		NVBP expansion (May 2020 – December 2021)	
			Level change β_2 (95% CI)	Slope change β_3 (95% CI)	Level change β_4 (95% CI)	Slope change β_5 (95% CI)
**Chronic IHD**						
Total	12817.66 (12025.28 to 13610.03)	-21.25 (-54.91 to 12.42)	893.68 (-24.04 to 1811.39)	-84.97 (-213.67 to 43.74)	920.91 (154.5 to 1687.32) *	46.77 (-94.99 to 188.53)
OOP	5008.29 (4731.85 to 5284.73)	-13 (-25.51 to -0.49) *	95.52 (-274.06 to 465.09)	-44.7 (-94.06 to 4.65)	472.71 (175.97 to 769.44) **	54.26 (0.01 to 108.5) *
Drug	2559.39 (2314.58 to 2804.21)	-3.11 (-14.41 to 8.18)	-49.53 (-343.5 to 244.44)	17.44 (-28.11 to 63)	-7.49 (-272.44 to 257.46)	-56.25 (-104.51 to -7.98) *
Diagnostic	3553.19 (3344.92 to 3761.45)	0.32 (-10.27 to 10.9)	-62.18 (-301.28 to 176.92)	-8.4 (-48.39 to 31.59)	-230.87 (-454.35 to -7.38) *	46.27 (4.41 to 88.14) *
Treat	2505.83 (2297.34 to 2714.32)	9.3 (0.19 to 18.4) *	85.68 (-177.92 to 349.28)	-36.46 (-73.48 to 0.57)	444.64 (221.89 to 667.4) ***	-2.93 (-43.75 to 37.9)
Consumable	641.28 (523.1 to 759.45)	4.26 (-1.19 to 9.71)	19.05 (-121.07 to 159.17)	-4.89 (-24.41 to 14.62)	-32.44 (-147.56 to 82.68)	15.93 (-5.28 to 37.14)
**Diabetes**						
Total	10602.93 (10294.06 to 10911.8)	-7.73 (-16.74 to 1.28)	-	-	-325.73 (-617.55 to -33.91) *	12.15 (-23.64 to 47.95)
OOP	3714.52 (3546.95 to 3882.08)	-11.73 (-14.39 to -9.06) ***	-	-	144.39 (46.64 to 242.14) **	22.61 (6.52 to 38.7) **
Drug	3084.55 (2864.13 to 3304.97)	-13.01 (-18.13 to -7.89) ***	-	-	-301.24 (-486.37 to -116.11) **	0.88 (-25.23 to 26.99)
Diagnostic	4036.31 (3905.73 to 4166.88)	0.07 (-3.82 to 3.95)	-	-	208.03 (77.71 to 338.35) **	0.95 (-12.9 to 14.81)
Treat	1272.54 (1176.99 to 1368.08)	0.09 (-1.75 to 1.93)	-	-	-48.87 (-121.05 to 23.31)	-1.66 (-13.5 to 10.18)
Consumable	530.06 (-7002.95 to 8063.07)	-2.66 (-10.51 to 5.2)	-	-	-56.35 (-119.72 to 7.03)	10.01 (-4.04 to 24.05)

The analysis of diabetes before the implementation of NVBP spanned from January 2017 to April 2020; OOP = out-of-pocket; Chronic IHD = chronic ischaemic heart disease; *** $p < 0.001$; ** $p<0.01$; * $p<0.05$

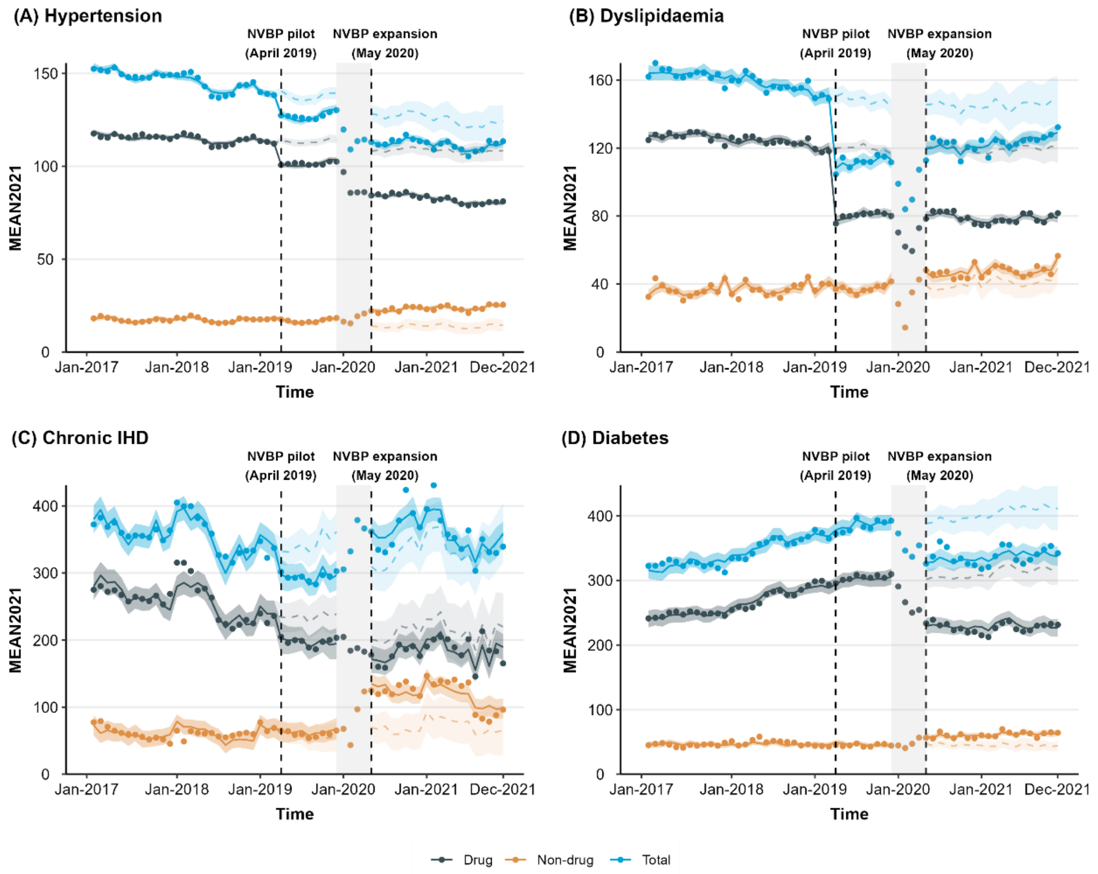

Figure S1. Interrupted Time Series plots of Outpatient Expenditures. Shown are the results of interrupted time-series analyses of the level and trend changes of outpatient expenditures per visit before the pilot of the NVBP program, after the pilot of NVBP program, and after the expansion of NVBP program. Panel (A) shows that outpatient expenditures for hypertension. Panel (B) shows the outpatient expenditures for dyslipidemia. Panel (C) shows the outpatient expenditures for chronic ischeamic heart disease. Panel (D) shows the outpatient expenditures for diabetes. The dots show the observed average expenditures for each month. The solid line represents the model-fitted expenditures estimated from the segmented generalized least-squares models accounting for the seasonality, time-varying confounder and autocorrelation. The dashed line represents the model-based expected expenditures assuming that the NVBP program had not been implemented (counterfactual). The shading indicates 95% confidence interval of the estimates. The gray rectangular shaded area represents the outbreak of COVID-19.

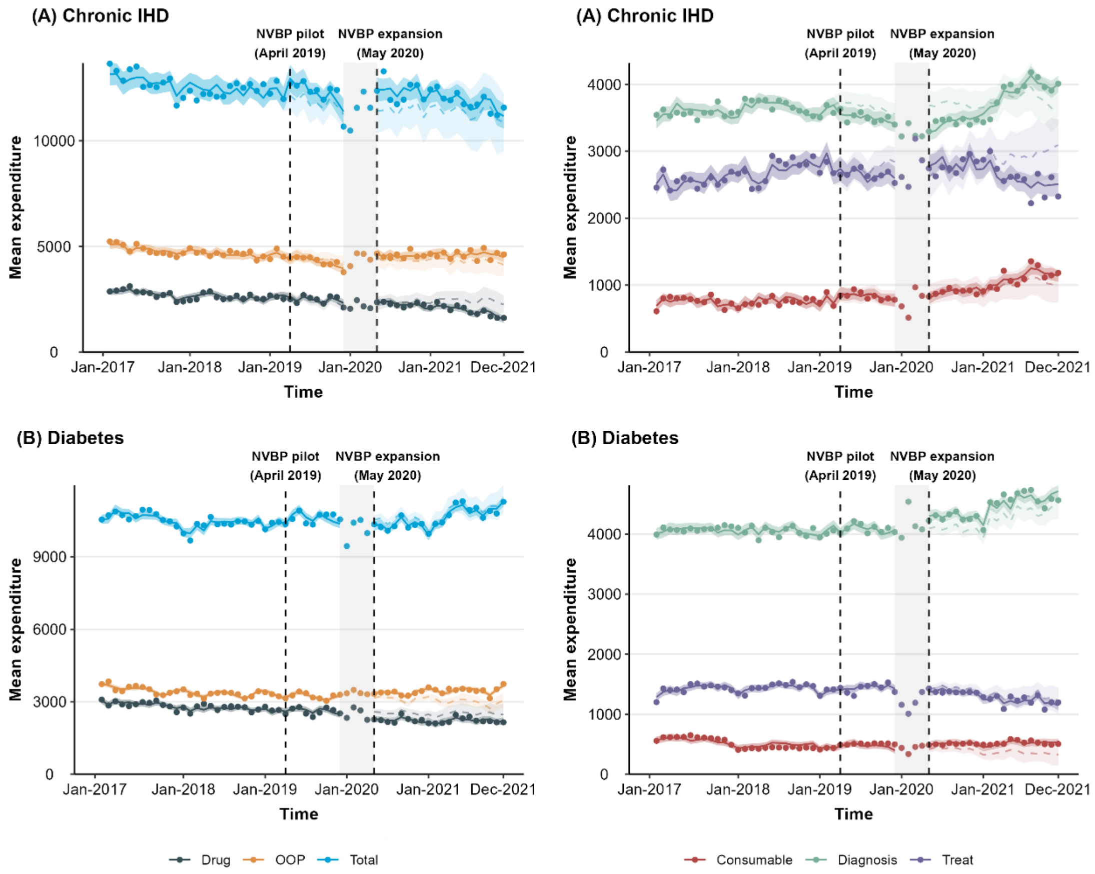

Figure S2. Interrupted Time Series Plots of Inpatient Expenditures. Shown are the results of interrupted time-series analyses of the level and trend changes of inpatient expenditures per admission. Chronic IHD = chronic ischeamic heart disease. OOP = out-of-pocket expenditures. Panel (A) shows the inpatient expenditures for chronic ischeamic heart disease. Panel (B) shows the inpatient expenditures for diabetes. The dots show the observed average expenditures for each month. The solid line represents the model-fitted expenditures estimated from the segmented generalized least-squares models accounting for the seasonality, potential time-varying confounder and autocorrelation. The dashed line represents the model-based expected expenditures assuming that the NVBP program had not been implemented (counterfactual). The shading indicates 95% confidence interval of the estimates. The gray rectangular shaded area represents the outbreak of COVID-19.

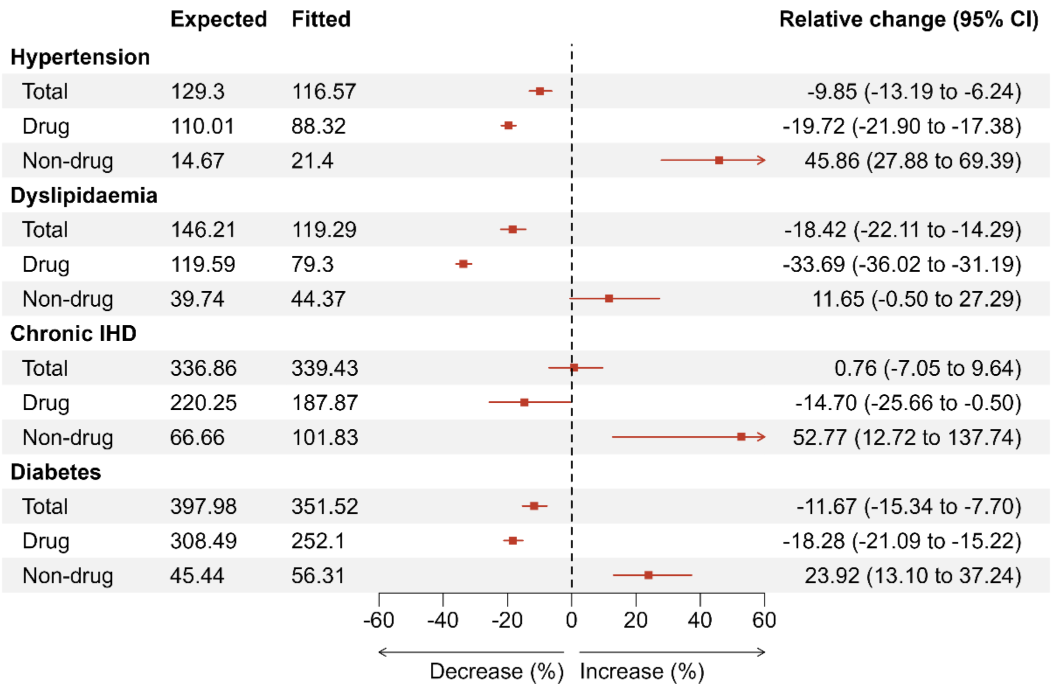

Figure S3. Relative Changes (%) in Outpatient Expenditures after NVBP. Shown are the estimated relative changes in outpatient expenditures per visit 2.75 years after the implementation of the NVBP program. Fitted values refer to the average values predicted by the segmented generalized least-squares models after the implementation of the NVBP program. Expected values represent the average values estimated from the segmented generalized least-squares models assuming that the NVBP program had not been implemented (counterfactual). The relative change is calculated as the difference between the fitted values and expected values, expressed as a proportion relative to the mean fitted values prior to the NVBP implementation. Squares indicate point estimates, and error bars indicate 95% confidence intervals calculated from 10,000 simulations. Chronic IHD = chronic ischaemic heart disease. Non-drugs were calculated as the total expenditures of outpatient care minus drug expenditures.

	Expected	Fitted	Relative change (95% CI)
**Chronic IHD**			
Total	11521.5	12114.46	5.15 (-3.08 to 14.84)
OOP	4355.42	4499.29	3.30 (-5.00 to 12.95)
Drug	2401.93	2201.6	-8.34 (-18.98 to 5.14)
Diagnosis	3659.67	3647.4	-0.34 (-7.41 to 8.01)
Treat	2839.11	2663.4	-6.19 (-14.55 to 3.93)
Consumable	925.07	963.74	4.18 (-11.48 to 26.16)
**Diabetes**			
Total	10734.4	10597.77	-1.27 (-3.53 to 1.11)
OOP	3103.55	3366.86	8.48 (5.26 to 12.00)
Drug	2554.34	2352.97	-7.88 (-13.42 to -1.75)
Diagnosis	4176.27	4326.64	3.60 (1.18 to 6.09)
Treat	1379.69	1333.97	-3.31 (-7.92 to 1.73)
Consumable	482.2	515.8	6.97 (-21.33 to 19.92)

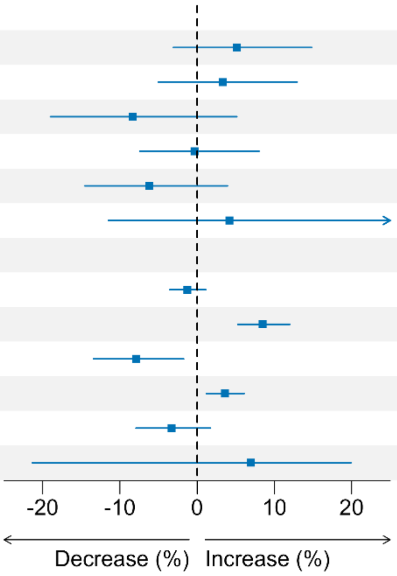

Figure S4. Relative Changes (%) in Inpatient Expenditures after NVBP. Shown are the estimated relative changes in inpatient expenditures per admission 2.75 years after the implementation of the NVBP program. Fitted values refer to the average values predicted by the segmented generalized least-squares models after the implementation of the NVBP program. Expected values represent the average values estimated from the segmented generalized least-squares models assuming that the NVBP program had not been implemented (counterfactual). The relative change is calculated as the difference between the fitted values and expected values, expressed as a proportion relative to the mean fitted values prior to the NVBP implementation. Squares indicate point estimates, and error bars indicate 95% confidence intervals calculated from 10,000 simulations. Chronic IHD = chronic ischaemic heart disease. OOP = out-of-pocket.
